# Recent Trends in Cigarette and HTP Use in Japan: A Scoping Review

**DOI:** 10.1093/ntr/ntaf216

**Published:** 2025-10-24

**Authors:** Mona Issabakhsh, Shannon Gravely, Kota Katanoda, Nargiz Travis, Radhika Ranganathan, Christopher J Cadham, Zhe Yuan, Yameng Li, Alex C Liber, Kenneth Michael Cummings, David T Levy

**Affiliations:** Lombardi Comprehensive Cancer Center, Georgetown University, 2115 Wisconsin Ave NW, Washington DC, 20007, United States; Department of Psychology, University of Waterloo, 200 University Avenue, West Waterloo, Ontario, N2L 3G1, Canada; Division of Population Data Science, National Cancer Center Institute for Cancer Control, 5-1-1 Tsukiji Chūō-ku, Tokyo, 104-0045, Japan; Lombardi Comprehensive Cancer Center, Georgetown University, 2115 Wisconsin Ave NW, Washington DC, 20007, United States; School of Business and Economics, RV University, RV Vidyanikethan Post, 8th Mile, Mysuru Road, Bengaluru, Karnataka, 560 059, India; Department of Health Management and Policy, School of Public Health, University of Michigan, 1415 Washington Heights, Ann Arbor, MI, 48109-2029, United States; Lombardi Comprehensive Cancer Center, Georgetown University, 2115 Wisconsin Ave NW, Washington DC, 20007, United States; Lombardi Comprehensive Cancer Center, Georgetown University, 2115 Wisconsin Ave NW, Washington DC, 20007, United States; Research Triangle Institute, Research Triangle Park, 3040 East Cornwallis Road, Durham, NC, 27713, United States; Hollings Cancer Center, Medical University of South Carolina, 86 Jonathan Lucas Street, MSC 955, Charleston, SC, 29425, United States; Lombardi Comprehensive Cancer Center, Georgetown University, 2115 Wisconsin Ave NW, Washington DC, 20007, United States

## Abstract

**Introduction:**

The emergence and rapid increase in sales and use of heated tobacco products (HTPs) in Japan provides a unique case study of their viability as a potentially lower-risk substitute for combustible cigarettes. This review investigates the relationship between HTP and cigarette use in Japan.

**Methods:**

We searched PubMed and Web of Science for studies on HTP and cigarette use, including sales trends, prevalence, and transitions between HTPs and cigarettes from 2010 to 2024. We distinguish results by source of funding and survey design.

**Results:**

Our review included 25 relevant studies, of which 21 reported HTP and/or cigarette prevalence and transitions and 4 reported sales trends. Cigarette sales and use rapidly declined during the national expansion of HTPs. HTP use increased substantially from 2015 to about 2019, then slowed through 2023. Trends from industry-sponsored studies were mostly in line with the government-sponsored estimates. Estimates from government-sponsored (mostly in-person) surveys indicate that cigarette use continuously declined from 2015 to 2023 as HTP growth increased, although at a slower pace since 2018. After decreasing cigarette prevalence from 2015 to 2018, online surveys reported high rates of dual cigarette-HTP use and comparatively low rates of smoking discontinuation from 2018 to 2023.

**Conclusions:**

The rapid decline in cigarette use from 2015 to 2018 in Japan suggests that increasing HTP use may have contributed to this trend. After 2018, slowing HTP sales and mixed estimates of cigarette and HTP use raise uncertainty about the role of HTPs. As such, the evidence remains incomplete, limiting definitive conclusions. The current study highlights the challenges associated with distinguishing the impact of HTPs on displacing cigarettes.

**Implications:**

This review provides evidence that HTP use likely contributed to declines in cigarette use in Japan from 2015 to 2018, though recent trends are less conclusive. It highlights differences across data sources and survey types, which can affect how results are interpreted. The study adds to our understanding of how HTPs may or may not replace cigarettes over time and points to the need for better, more consistent data to track these trends.

## Introduction

The sale and use of heated tobacco products (HTPs) rapidly increased in Japan^1–4^ and the Republic of Korea.^5–8^ HTPs, particularly IQOS, introduced by Philip Morris International (PMI),^9^ have also risen in popularity in other countries, including some European countries (eg, Poland and Italy).^10–21^ Like e-cigarettes, HTPs are designed with the intent of inhalation and have similar sensorimotor experiences and “throat-hit” to conventional cigarettes, which is achieved by heating tobacco leaves rather than vaporizing a nicotine solution.^22–24^

Even though HTPs have been available for decades (since the 1980s in the USA), no long-term epidemiologic studies have assessed their overall harm-reduction benefits.^25^ Projected health benefits from HTPs are based on reduced smoke emissions and toxicology relative to cigarettes.^26–32^ Public health may be improved when HTPs completely replace cigarette use by those who previously smoked.^33^ However, HTPs may worsen health if used by those who did not smoke or those who continue to smoke cigarettes (ie, “dual-use”) instead of entirely switching to HTPs or no use.^33^

An early review of the effect of HTPs in different countries found limited evidence of their impact on cigarette use.^32^ A more recent review concluded that HTPs may be a gateway to and may discourage cessation from cigarette use.^34^ However, that review included studies only through 2022 and combined results from countries with different regulatory approaches to potential harm-reduction products.^21^ For example, HTP use in Japan, where e-cigarettes are not legally sold in the domestic market, and Korea, where e-cigarettes are legally sold, may be differentially affected by e-cigarette use. They included 17 studies for Japan but inexplicitly excluded many studies, including those examining sales trends.^3^^,^^35–37^

As a particularly dynamic HTP market,^38^ Japan provides a unique opportunity to study its impact. PMI introduced IQOS in Nagoya, Japan, in 2014, with national expansion in 2015.^39^ Other HTP brands were introduced by British American Tobacco (BAT) in 2016 and Japan Tobacco International (JTI) in 2017.^3^^,^^15^^,^^17^^,^^35^ This study reviews the published literature on HTP and cigarette sales and use in Japan. We focus on evidence examining whether HTPs have replaced or supplemented cigarette use and distinguish results by source of funding and by survey design.

## Methods

### Overview

Our review considers cigarette and HTP sales, prevalence, and transitions in Japan. Because HTPs are a relatively new product without well-defined use measures and with a potentially complex relationship to cigarette use, we conducted a scoping review,^40^ rather than a systematic review. Our review follows the Preferred Reporting Items for Systematic Reviews and Meta-Analyses extension for Scoping Reviews (PRISMA-ScR) guidelines and was pre-registered on the Open Science Framework (OSF).^41^

### Data Sources, Searches, and Study Selection

Our literature search was conducted on December 31, 2024 in PubMed and Web of Science by two reviewers (MI, RR). We used the search terms “Japan” AND the following connected by OR (“heated tobacco,” “HTP,” “heat not burn,” “IQOS,” “Glo,” “Ploom,” “tobacco product,” “cigarette,” “smoking,”) AND the following connected by OR (“sale,” “trend,” “transition,” “prevalence,” “pattern,” “change,” “shift,” “evolution,” “switch,” “initiation,” “cessation,” “quit,” “use”).

Studies were limited to those that reported cigarette or HTP sales trends, prevalence, or behavioral transitions, such as initiation, cessation, or switching rates between the two products. The year 2010 was selected as the start year of our review because smoking prevalence and tobacco control policies in Japan were relatively stable from 2010 to 2014.^42^^,^^43^ We began our search in 2010 to capture smoking trends prior to HTPs entering the market and included studies published in English through December 31, 2024. We distinguish 2010–2015 as the “pre-HTP period” (before HTPs’ national expansion in 2015)^3^ and 2015–2024 as the “post-HTP period.” Studies were included if the authors clearly stated that they measure country-level sales or that they use a sampling method designed to estimate population-level HTP use and cigarette smoking. Clinical trials, modeling and simulation studies, qualitative studies, non-nationally representative studies, and studies in countries other than Japan were excluded. While we included studies that analyzed participants of specific age groups (starting from age 15), we excluded papers considering only specific sub-populations (eg, cigarette and HTP use among retail business workers).

**Figure 1:**
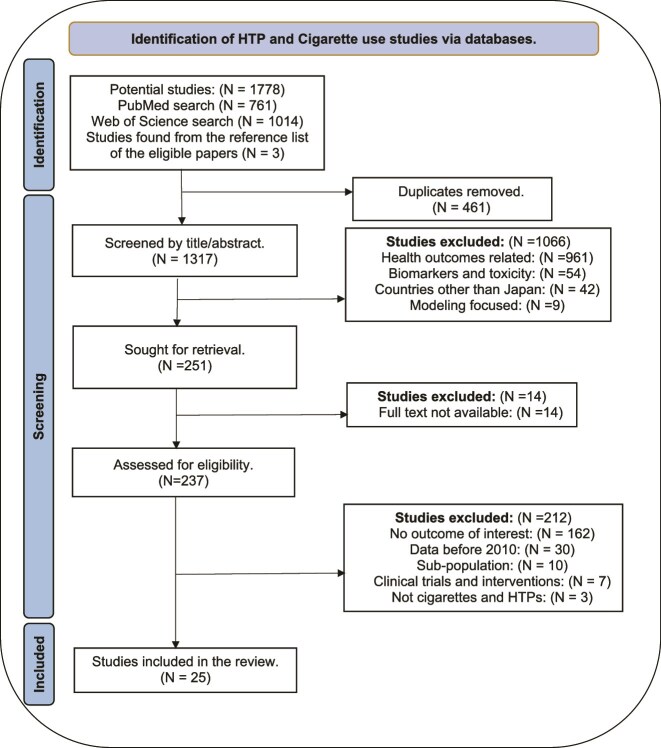
A PRISMA flow diagram of study selection.

Two reviewers (MI, RR) extracted and screened titles and abstracts, using Rayyan, a web-based systematic review tool.^44^ Reviewers were blinded to authors’ names and affiliations and journals of publication. They independently screened full-text articles for eligibility criteria and searched reference lists of eligible papers for additional sources. Any reviewer disagreements were resolved by a third reviewer (DTL). Figure 1 provides a PRISMA flow diagram of the study selection process. The complete search strategy and a list of excluded studies that received a full-text read are available in Supplement 1.

### Data Extraction and Synthesis

The reviewers extracted study-specific information (study type [longitudinal vs. cross-sectional], survey type [eg, household survey, online panel], other study design features [eg, the data source, sample size, data collection time, tobacco use definitions, and age groups], outcome types and results). We relied on the text of the reviewed studies to extract the study-specific information. For instance, if a study clearly stated it was “nationally representative” or “nationwide,” we classified it as nationally representative. The extracted results were limited to cigarettes and HTPs’ sales volume, prevalence (including total, exclusive, and dual-use), and initiation, cessation, and switching rates.

We categorized studies by: (1) cigarette use in terms of sales and prevalence (including overall cigarette, exclusive cigarette, and dual cigarette-HTP), (2) HTP use in terms of sales and prevalence (overall, exclusive, and dual), and (3) transitions in terms of initiation, cessation, relapse and product switching involving cigarette and HTP use. We distinguish cross-sectional/age-period-cohort (APC) and longitudinal-based studies within each category. We also compare the consistency of results between cigarette industry-sponsored and non-industry-sponsored studies and compare results from major surveys in Japan.

## Results

As shown in Figure 1, our broad search of keyword terms identified 1778 studies, of which we assessed 237 for eligibility and included 25 that met the inclusion criteria. Our results included 21 prevalence and transition (11 cross-sectional/APC, 10 longitudinal) and 4 sales analysis studies. Eighteen studies reported outcomes for both cigarettes and HTPs, 3 only for cigarettes, and 4 only for HTPs.

Our search also identified major national tobacco use surveys in Japan. Two government-sponsored, primarily in-person surveys (the National Health and Nutrition Survey [NHNS],^45^ and the Comprehensive Survey of Living Conditions [CSLC]^46^) have been conducted over 30+ years and use probability sampling. In this review, any data or analysis involving NHNS and CSLC are considered “government-sponsored.”

The Japan “Society and New Tobacco” Internet Survey (JASTIS)^47^ and the International Tobacco Control (ITC)^48^ are web-based surveys that apply a nonprobability sampling method from a major online panel, herein referenced as “online studies.” The JASTIS is a longitudinal internet cohort survey conducted since 2015. JASTIS studies include both longitudinal cohorts and cross-sectional samples. For comparability, all estimates from JASTIS are discussed together in the “Longitudinal-Based” sub-sections of this paper. Of note, industry-sponsored research includes separate studies using in-person and online sampling.

### Cigarette Use

Four studies reported sales trends, while 15 reported cigarette and dual cigarette-HTP prevalence (9 cross-sectional and APC, 6 longitudinal). Table 1 presents the characteristics of studies that reported cigarette prevalence, and Table S1 of Supplement 2 presents the total and exclusive cigarette and dual cigarette-HTP prevalence from studies. “Cigarette prevalence” below refers to total cigarette prevalence as distinct from exclusive cigarette prevalence. We also consider the percentage of dual cigarette-HTP use among those smoking cigarettes, as stated or inferred from the reported rates.

**Table 1:** Properties of the Studies that Reported Cigarette and Dual use Prevalence

**Study**	**Data source**	**Nationally representative?**	**Cross-sectional/APC or longitudinal**	**Survey type**	**Years conducted**	**Sample size**	**Age**	**Tobacco use definition**
Okui^49^	NHNS	Yes	APC	In-person	1995–2018	6544 (2018)	≥20	Every day or some days (2018)
Levy et al.^50^	NHNS	Yes	Cross-sectional	Online	2011–2019	5709 (2019)	≥20	Every day or some days (2019)
Okui^53^	CSLC	Yes	APC	In-person	2004–2019	720 000 (2019)	≥20	Every day or some days
Kuwabara et al.^55^^,^^56^	LSA	Yes	Cross-sectional	In-person	2017	64 152	15–18	Past-30-day
Sutanto et al.^57^	ITC	Yes	Cross-sectional	Online	2018	4684	≥20	At least monthly
Afolalu et al.^58^^*^	***PMI/JGAP***	Yes	Cross-sectional	In-person	2016–2017	4878	≥20	At least monthly use
Afolalu et al.^58^^*^	***PMI/JAIQOS***	No	Cross-sectional	Online	2016–2017	2000	≥20	At least monthly use with at least 100 times use lifetime
Fischer et al.^59^^*^	***PMI/1)JGAP and 2)JAIQOS***	1) Yes and 2) No	Cross-sectional (three periods)	1) In-person and 2) Online	2016–2019	1) 5000 and 2) 2000 per year	≥20	Daily or non-daily use, with at least 100 times use lifetime
Jones et al.^60^	***BAT***	Yes	Cross-sectional	In-person	2019	5306	≥20	At least 100 times use lifetime
Odani et al.^61^	JASTIS	Yes	Longitudinal (three time points, 2019 baseline, 2020 follow-up, and 2020 baseline, 2021 follow-up)	Online	2019–2021	7044	≥20	Past-30-day
Sugiyama and Tabuchi^4^^*^	JASTIS	Yes	Cross-sectional sample	Online	2017	10 114	≥15	Past-30-day
Odani and Tabuchi^17^	JASTIS	Yes	Cross-sectional sample	Online	2020	9044	≥15	Past-30-day
Odani and Tabuchi^62^	JASTIS	Yes	Cross-sectional sample	Online	2022	28 124	≥16	Past-30-day
Odani and Tabuchi^63^	JASTIS	Yes	Cross-sectional sample	Online	2023	29 354	≥16	Past-30-day
Yamamoto et al.^64^^*^	JASTIS	Yes	Cross-sectional sample	Online	2022	30 141	≥17	Past-30-day

***Bolded Italicized*** studies are Industry-sponsored. ^*^Exclusive cigarette prevalence means “only cigarette use and no other tobacco use,” and dual cigarette-HTP prevalence means “only dual cigarette-HTP use and no other tobacco use.”

#### Sales Trends

Using 2014–2018 monthly retailer panel data, Stoklosa et al.^3^ found that cigarette sales began to decline when IQOS was introduced in different areas of the country. Using 2011–2020 Tobacco Institute of Japan (TIJ) cigarette sales and PMI HTP sales data, Pesola et al.^36^ reported that cigarette sales decreased as HTP sales increased. Extending an earlier study^35^ and using PMI data, Cummings et al.^37^ reported that cigarette sales declined by 52.7% from 2011 to 2023. The annual percentage change in per capita cigarette sales was −1.5% from 2011 to 2015, which accelerated to −10.5% in 2015–2018 during the period of rapid HTP growth, and continued to fall at −7.3% from 2018 to 2023 as HTP growth declined. Sales analyses do not distinguish the role of cigarette prices, prevalence versus quantity purchased, or dual cigarette-HTP versus exclusive cigarette use.

#### Cross-Sectional and APC Studies

In an APC analysis of daily and non-daily cigarette prevalence using the 1995–2018 NHNS, Okui^49^ (ages ≥20) found that male (female) cigarette prevalence increased by 3.7% (2.6%) from 2010 to 2014, dropped by 12.8% (23.1%) from 2014 to 2017, and by 15.1% (9.4%) between 2014 and 2018.

Using NHNS, Levy et al.^50^ (ages ≥20) found accelerating declines in male and female cigarette prevalence in 2014–2017 relative to 2011–2014. The accelerated decline was most pronounced for males aged 20–49, the ages with the highest reported HTP use. Since that study, NHNS data have become available for 2022 and 2023,^51^^,^^52^ with overall cigarette prevalence falling from 18% in 2018 to 13% in 2019 to 11% in 2022 and 2023. The percentage of dual-use among those who smoke cigarettes decreased from 11% in 2018 to 8% in 2019 to 6% in 2022, then increased to 13% in 2023. However, Levy et al.^50^ identified measurement issues from frequent changes in the format and wording of NHNS questions.^45^ Notably, NHNS changed the format of questions in 2018, distinguishing “smoking” products by asking specifically about cigarettes, HTPs, and other types of tobacco use, yielding a substantial drop in the estimated smoking prevalence between 2017 and 2018.

Conducting an APC analysis using the 2004–2019 CSLC, Okui^53^ (ages ≥20) reported that urban (non-urban) cigarette prevalence among men increased by 4.4% (3.3%) from 2010 to 2013, decreased by 9.1% (4.3%) from 2013 to 2016, and by 4.3% (6.0%) between 2016 and 2019, and among women increased by 2.5% (6.7%) between 2010 and 2013, decreased by 12.4% (6.3%) between 2013 and 2016 and by 4.7% (6.7%) from 2016 to 2019. More recent CSLC data^54^ indicate that male (female) cigarette prevalence fell from 28.8% (8.8%) in 2019 to 25.4% (7.7%) in 2022, a relative decline of about 11.8% (12.5%). Notably, the CSLC specifically asks, “How many cigarettes do you smoke on average per day?” thereby associating smoking directly with cigarette use.^54^

Using the 2017 Lifestyle Survey of Adolescents (LSA), Kuwabara et al.^55^^,^^56^ (ages 15–18) reported 1.5% cigarette prevalence and 46% dual-use among those smoking cigarettes.

Analyzing a sample of cigarette, HTP, and non-tobacco users from the 2018 Japan ITC survey, Sutanto et al.^57^ (ages ≥20) reported 8.8% at least monthly dual cigarette-HTP use among those smoking cigarettes.

Three industry-sponsored studies reported cigarette prevalence. Extending an earlier PMI study,^58^ Fischer et al.^59^ (ages ≥20) reported on two cross-sectional surveys: an in-person representative sample of adults (JGAP) and an online and non-nationally representative sample of adult IQOS users (JAIQOS) from PMI’s internet database from 2016/17 to 2018/19. Over the 3 years, cigarette prevalence from JGAP decreased from 17.6% to 17.3% to 16.0%, with implied 5%, 9%, and 7% yearly dual-use among those smoking cigarettes. Dual IQOS-cigarette users in JGAP are those who only use IQOS and cigarettes, excluding participants who use other types of tobacco (eg, e-cigarettes). Applying a sample of those who only use IQOS, the prevalence of dual IQOS and combustible tobacco use from JAIQOS decreased from 28.4% to 25.4% to 23.6%. Of note, JAIQOS results are susceptible to bias since participants were past IQOS purchasers and not representative of the general population.

In a 2019 BAT-sponsored study, Jones et al.^60^ (ages ≥20) reported 14.6% cigarette prevalence, with 16% implied dual-use among those smoking cigarettes.

#### Longitudinal-Based Studies

All studies in this section reported data from the JASTIS.^47^ A longitudinal analysis by Odani et al.^61^ (ages ≥20) reported total cigarette prevalence of 16.4% (10% exclusive) in 2019 and 18.6% (12.7% exclusive) in 2020 (13% relative increase). Dual cigarette-HTP prevalence decreased from 6.4% in 2019 to 5.9% in 2020 (an 8% relative reduction). The implied percentage of dual-use among those who smoke cigarettes was 39% in 2019 and 32% in 2020.

Other studies analyzed cross-sectional samples (presented for a single time point) from JASTIS. Sugiyama and Tabuchi^4^ (ages ≥15) reported 20.1% cigarettes and 1.6% dual cigarette-HTP prevalence (8% of those smoking) in 2017. Dual-users were defined as those who only used cigarettes and HTPs, excluding those who used other types of tobacco (eg, e-cigarettes). Odani and Tabuchi^17^ (ages ≥15) reported 25.9% cigarette and 8.6% dual cigarette-HTP prevalence in 2020, implying 33% dual-use among those who smoke cigarettes. Odani and Tabuchi^62^ (ages ≥16) reported 19.4% cigarette (12.6% exclusive) and 6.8% dual combustible-HTP prevalence in 2022, implying 35% dual-use. For 2023, Odani and Tabuchi^63^ (ages ≥16) reported 18.9% (27.6% men; 10.4% women) cigarette prevalence and 7.4% dual prevalence (11.6% men; 3.4% women), implying 39% dual-use. For 2022, Yamamoto et al.^64^ (ages ≥17) reported 14% cigarette and 3.2% dual cigarette-HTP prevalence (23% among those who smoke cigarettes). Exclusive cigarette use was highest among ages 50–59, while dual cigarette-HTP use was highest among ages 40–49. Those who smoked cigarettes and used any other type of tobacco were excluded from exclusive cigarette and dual cigarette-HTP use.

#### Summary

Figure 2A summarizes the results from the different studies and surveys for total (dual and exclusive) cigarette prevalence. A flat trend is seen in cigarette prevalence from 2010 to 2015, followed by a downward trend through 2023. Point estimates vary between studies, especially after 2018. Data from NHNS and CSLC indicate declining trends from 2013 to 2022. Compared to studies not funded by the tobacco industry, industry-sponsored studies report slightly lower cigarette prevalence in 2016 and 2017 but higher prevalence in 2018 than that of the NHNS and CSLC. Meanwhile, online JASTIS studies report a slightly declining rate of cigarette prevalence from 2017 to 2019 but increasing prevalence through 2020 and remaining relatively constant through 2023. However, anomalous results were reported in JASTIS 2020,^17^^,^^61^ with one study cross-sectional^17^ and the other longitudinal.^61^ Anomalous results were also reported in JASTIS 2022,^62^^,^^64^ with one study appearing to exclude other types of nicotine-delivery products (eg, e-cigarettes) from exclusive cigarette and dual-use.^64^ Cross-sectional JASTIS studies had sample sizes of ~10 000 in 2017–2020,^4^^,^^17^ increasing to ~30 000 in 2022–2023.^62^^,^^63^ Comparatively, longitudinal JASTIS studies had smaller sample sizes of ~5000 in 2015–2016,^65^ ~8000 in 2015–2017,^66^ and ~7000 in 2019–2021.^61^ Age ranges in JASTIS studies also varied from ≥15^4^ to ≥20.^61^ Not shown, sales data analyses reported declining cigarette use through 2023, with less decline since 2018.^3^^,^^35–37^

**Figure 2:**
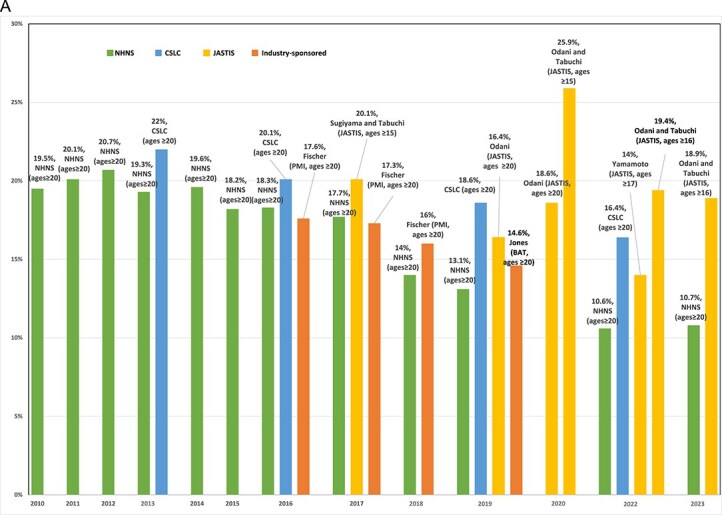
(A) Total cigarette prevalence. Notes: (1) Since 2018, the NHNS has included a question about the types of tobacco products used, and the cigarette prevalence has been calculated by multiplying the overall tobacco prevalence by the proportion of total cigarette use. (2) In the Yamamoto study in 2022, cigarette users who used any other type of tobacco (eg, e-cigarettes) were excluded from exclusive cigarettes and dual-use. The total cigarette prevalence is calculated as the summation of exclusive and dual cigarette, which resulted in lower total cigarette prevalence. (3) Fischer reported IQOS prevalence, not all HTPs. (4) Different tobacco use definitions are considered by studies, which should be taken into account when interpreting the figure. For more information on tobacco use definitions, see Tables 1 and 2.

Figure 2B presents the percentage of dual-use among those who smoke cigarettes. Fischer et al.,^59^ Sugiyama and Tabuchi,^4^ and Yamamoto et al.^64^ defined dual cigarette-HTP use, excluding participants who used other types of tobacco and reported lower rates of dual-use among those who smoked cigarettes. Cigarette industry-sponsored studies had similar percentages of dual-use to the 2018–2019 NHNS (although only including those using IQOS in PMI studies). A similar percentage of dual-use is reported in 2017 by Fischer et al.^59^ (PMI) and Sugiyama and Tabuchi^4^ (JASTIS), but a lower percentage of dual use is reported in 2018 by Fischer et al.^59^ compared to non-industry-funded studies. Further, the percentage of dual use reported in 2019 by Jones et al.^60^ (BAT) is higher than that of NHNS and lower than that of JASTIS. In general, the percentage of dual-use reported in JASTIS and ITC accelerated from 9% in 2018 to 40% in 2023.

**Figure 2B:**
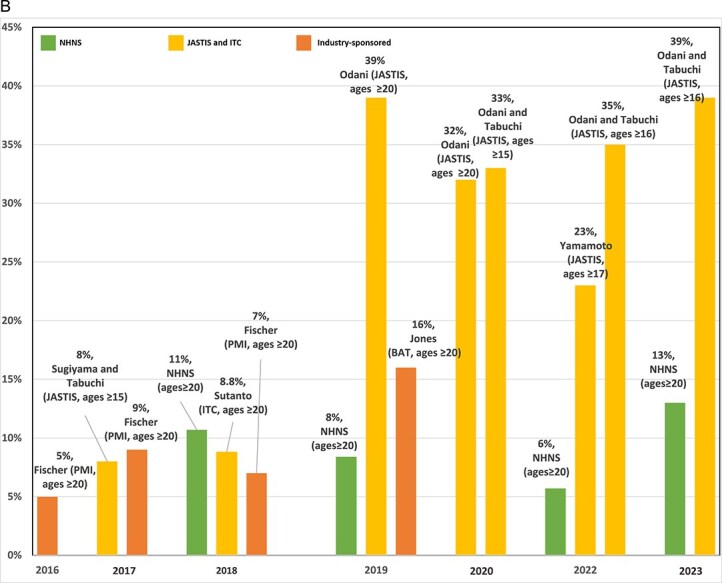
Percentage of dual-use among those who smoke cigarettes. Notes: (1) If not reported by the studies, the percentage of dual use among those using cigarettes is inferred as (the prevalence of dual use/total cigarette prevalence) ^*^ 100. (2) In the NHNS, the percentage of dual use among those using cigarettes is calculated as (the proportion of dual use among tobacco users/the proportion of total cigarette use among tobacco users) ^*^ 100. (3) The following studies defined dual cigarette-HTP use excluding participants who used other tobacco, and therefore, reported a lower rate of dual use: Fischer 2016, 2017, 2018; Sugiyama and Tabuchi, 2017; Yamamoto 2022. (4) Fischer reported IQOS prevalence, not all HTPs; (5) different tobacco use definitions are considered by studies, which should be taken into account when interpreting the figure. For more information on tobacco use definitions, see Tables 1 and 2.

### HTP Use

Seventeen studies (8 cross-sectional and 9 longitudinal) reported HTP prevalence. Table 2 presents the characteristics of these studies, and Table S2 of Supplement 2 presents the prevalence of HTPs in reviewed studies. “HTP prevalence” below refers to total HTP as distinct from “exclusive HTP prevalence.” We also consider the percentage of dual cigarette-HTP use among those who use HTPs as stated or inferred from the reported prevalence rates.

**Table 2:** Properties of the Studies that Reported HTP Prevalence and use Rates

**Study**	**Data source**	**Nationally representative?**	**Cross-sectional or longitudinal?**	**Survey type**	**Years conducted**	**Sample size**	**Age**	**Current tobacco use definition**
Kinjo et al.^2^	PBSHTS	Yes	Cross-sectional	In-person	2018	4628	≥20	Past-30-day
Kuwabara et al.^55^^,^^56^	LSA	Yes	Cross-sectional	In-person	2017	64 152	15–18	Past-30-day
Sutanto et al.^67^	ITC	Yes	Cross-sectional	Online	2018	4684	≥20	At least monthly
Sutanto et al.^57^	ITC	Yes	Cross-sectional	Online	2018	4684	≥20	At least monthly
Afolalu et al.^58^^*^	***PMI/JGAP***	Yes	Cross-sectional	In-person	2016–2017	4878	≥20	At least monthly use
Afolalu et al.^58^^*^	***PMI/JAIQOS***	No	Cross-sectional	Online	2016–2017	2000	≥20	At least monthly use with at least 100 times use lifetime
Fischer et al.^59^^*^	***PMI/1)JGAP and 2)JAIQOS***	1)Yes and 2)No	Cross-sectional (three periods)	1) In-person and 2) Online	2016–2019	1) 5000 and 2) 2000 per year	≥20	Daily or non-daily use, with at least 100 times use lifetime
Jones et al.^60^	***BAT***	Yes	Cross-sectional	In-person	2019	5306	≥20	At least 100 times use lifetime
Tabuchi et al.^66^	JASTIS	Yes	Longitudinal (three time points, 2015 baseline, 2016 and 2017 follow-ups)	Online	2015–17	8240 (2015)	≥15	Past-30-day
Hori, Tabuchi, and Kunugita^65^	JASTIS	Yes	Longitudinal (two periods)	Online	2015–2016 and 2017–2018	5366 and 3422	≥15	Past-30-day
Hori, Tabuchi, and Kunugita^15^	JASTIS	Yes	Longitudinal (five time periods)	Online	2015–2019	8240 (2015)	≥15	Past-30-day
Odani et al.^61^	JASTIS	Yes	Longitudinal (three time points, 2019 baseline, 2020 follow-up, and 2020 baseline, 2021 follow-up)	Online	2019–2021	7044	≥20	Past-30-day
Sugiyama and Tabuchi^4^^*^	JASTIS	Yes	Cross-sectional sample	Online	2017	10 114	≥15	Past-30-day
Odani and Tabuchi^17^	JASTIS	Yes	Cross-sectional sample	Online	2020	9044	≥15	Past-30-day
Odani and Tabuchi^62^	JASTIS	Yes	Cross-sectional sample	Online	2022	28 124	≥16	Past-30-day
Odani and Tabuchi^63^	JASTIS	Yes	Cross-sectional sample	Online	2023	29 354	≥16	Past-30-day
Yamamoto et al.^64^^*^	JASTIS	Yes	Cross-sectional sample	Online	2022	30 141	≥17	Past-30-day

***Bolded italicized*** studies are Industry-sponsored.

^*^Exclusive HTP and dual cigarette-HTP prevalence means “only HTP use and no other tobacco use.”

#### Cross-Sectional Studies

Using the 2018 Population-Based Survey of Heated Tobacco Smoking (PBSHTS), Kinjo et al.^2^ (ages ≥20) reported that almost one-third (one-fifth) of males (females) who used tobacco used HTPs in the past month, with 7.3% (1.8%) HTP prevalence. Using the 2017 LSA, Kuwabara et al.^55^^,^^56^ (ages 15–18) reported 0.9% HTP prevalence (1.2% men; 0.6% women), with 70% dual-use among those using HTPs.

Among ITC studies conducted in 2018 (ages ≥20), Sutanto et al.^67^ reported an overall monthly and daily HTP prevalence of 2.7% and 1.7%, with 68%, 25%, and 1% HTP use among those who currently, formerly, and never smoked, respectively. Daily HTP use was more common among those who exclusively used HTPs, and dual-users mostly reported weekly and monthly HTP use. Sutanto et al.^57^ reported 63% at least monthly dual-use among those who use HTPs.

Among tobacco industry-sponsored studies, Fischer et al.^59^ (ages ≥20) reported that, among all HTP brands in JGAP, IQOS had the highest prevalence. In JGAP, IQOS prevalence increased from 1.8% in 2016/17 to 3.3% in 2018/19, while the percentage of dual-use among those who use HTPs decreased from 64% to 48%. Dual IQOS-cigarette use excluded participants who use other types of tobacco (eg, e-cigarettes). Applying a sample of only those who use IQOS, exclusive IQOS prevalence in JAIQOS decreased from 63.4% in 2016/17 to 49.4% in 2018/19. However, the prevalence of IQOS use with other smoke-free tobacco increased over the three years. A BAT-sponsored study^60^ (ages ≥20) reported 5.3% HTP prevalence in 2019, with 51% implied dual-use among those who use HTPs.

#### Longitudinal-Based Studies

All studies in this section reported data from the JASTIS.^47^ Using longitudinal JASTIS data, Tabuchi et al.^66^ (ages ≥15) reported that IQOS prevalence increased from 0.3% to 3.6% from 2015 to 2017. Hori, Tabuchi, and Kunugita^65^ (ages ≥15) reported that HTP prevalence increased from 0.7% in 2015–2016 to 5.6% in 2017–2018. Hori, Tabuchi, and Kunugita^15^ (ages ≥15) also reported that HTP prevalence increased from 0.2% in 2015 to 11.3% in 2019, with the 2019 HTP prevalence >30% among those who currently smoked cigarettes at baseline. Odani et al.^61^ (ages ≥20) reported HTP prevalence at 9.5% in 2019 and 8.5% in 2020, with implied dual-use at 67% in 2019 and 69% in 2020.

Other studies analyzed cross-sectional samples (presented for a single time point) from JASTIS. Sugiyama and Tabuchi^4^ (ages ≥15) reported 1.1% exclusive HTP and 1.6% dual cigarette-HTP prevalence in 2017, implying 59% dual-use among those who use HTPs. Those using HTPs who used other types of tobacco (eg, e-cigarettes) were excluded from exclusive HTP and dual-use prevalence. Odani and Tabuchi^17^ (ages ≥15) reported 10.9% HTP prevalence in 2020, of which 79% was dual-use. Among those who currently smoke cigarettes, 34.9% intended and 30.5% did not intend to quit cigarettes using HTPs (although not significantly different). Older adults were less likely to use HTPs than those aged 20–29. Odani and Tabuchi^62^ (ages ≥16) reported 11.8% HTP prevalence in 2022, implying 58% dual-use. Odani and Tabuchi^63^ (ages ≥16) reported 12.4% HTP prevalence in 2023, with 60% dual-use among those using HTPs. Yamamoto et al.^64^ (ages ≥17) reported 4.7% exclusive HTP and 3.2% dual cigarette-HTP prevalence in 2022, implying 41% dual-use among those using HTPs. However, those using HTPs who used other types of tobacco (eg, e-cigarettes) were excluded from exclusive HTP and dual-use prevalence.

#### Summary

Figure 3A summarizes the results from the different studies regarding total (dual and exclusive) HTP prevalence. Point estimates vary across studies (especially in 2018 and after), but the general trend appears consistent, with HTPs increasing markedly in 2016 and 2017. Online survey studies indicate that HTP prevalence increased substantially from 2015 to 2019,^66^ then grew more slowly since 2020.^4^^,^^15^^,^^17^ NHNS data indicate a higher HTP prevalence in 2018 but a lower prevalence and less growth in HTP use than online studies through 2023. Compared to studies not funded by the tobacco industry, similar HTP prevalence is reported for 2017 and 2018, but a lower HTP prevalence is reported in 2019 by industry-funded studies. Cigarette and HTP sales trends mirror the survey data.^37^

**Figure 3A:**
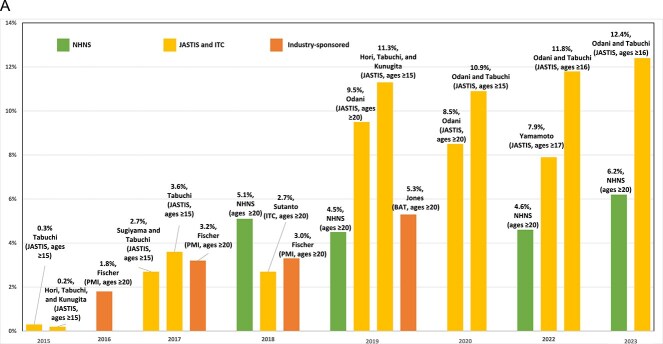
Total HTP prevalence. Notes: (1) Since 2018, the NHNS has included a question about the types of tobacco products used, and the HTP prevalence is calculated by multiplying the overall tobacco prevalence by the proportion of total HTP use. (2) The following studies defined exclusive HTP, and dual cigarette HTP use excluding participants who used other tobacco, and therefore, reported a lower rate of HTP prevalence: Sugiyama and Tabuchi, 2017; Yamamoto 2022. (3) Fischer reported IQOS prevalence, not all HTPs. (4) Different tobacco use definitions are considered by studies, which should be taken into account when interpreting the figure. For more information on tobacco use definitions, see Tables 1 and 2.

Figure 3B presents the percentage of dual-use among those using HTPs. Cigarette-industry-sponsored studies found 60% dual-use among those using HTPs in 2016–2017, falling to 50% in 2018–2019. NHNS estimates had a lower percentage of dual-use (30% in 2018), declining to 13% in 2022 but increasing to 23% in 2023. A similar percentage of dual-use is reported in 2017 by Fischer et al.,^59^ (PMI) and Sugiyama and Tabuchi^4^ (JASTIS). The percentage of dual use reported in 2018 and 2019 by industry-sponsored studies, however, is higher than that of NHNS and lower than that of JASTIS. Online studies indicate that the percentage of dual-use increased from 59% in 2017 to 67% in 2019, remained relatively constant through 2020, and then fell to about 60% through 2023. An exceptionally high percentage of dual-use (79%) was obtained by Odani and Tabuchi^17^ in 2020, and a low percentage (41%) by Yamamoto et al.^64^ in 2022. Fischer et al.,^59^ Sugiyama and Tabuchi,^4^ and Yamamoto et al.^64^ defined exclusive HTP and dual cigarette-HTP use, excluding participants who used other tobacco (eg, e-cigarettes), and thus their estimates are not directly comparable to other studies.

**Figure 3B:**
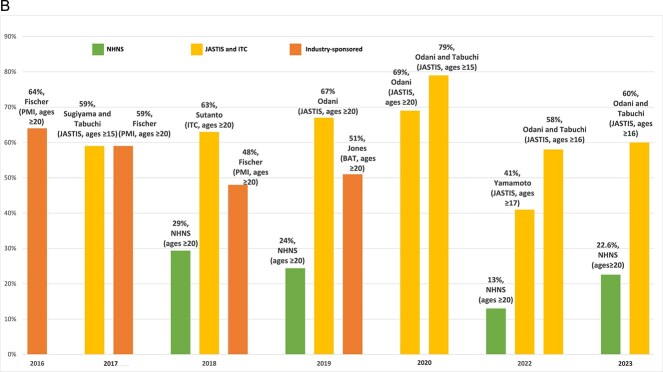
Percentage of dual-use among those using HTPs. Notes: (1) Since 2018, the NHNS has included a question about the types of tobacco products used, and the HTP prevalence is calculated by multiplying the overall tobacco prevalence by the proportion of total HTP use. The following studies defined exclusive HTP, and dual cigarette-HTP use excluding participants who used other tobacco, and therefore, reported lower rate of HTP prevalence: Sugiyama and Tabuchi, 2017; Yamamoto 2022. Fischer reported IQOS prevalence, not all HTPs. (2) If not reported by the studies, the percentage of dual use among those using HTPs is inferred as (the prevalence of dual use/total HTP prevalence) ^*^ 100. (3) In NHNS, the percentage of dual use among those using HTPs is calculated as (the proportion of dual use among tobacco users/the proportion of total HTP use among tobacco users) ^*^ 100. (4) The following studies defined exclusive HTP and dual cigarette-HTP use excluding participants who used other tobacco: Fischer 2016, 2017, 2018; Sugiyama and Tabuchi, 2017; Yamamoto 2022. (5) Fischer reported IQOS prevalence, not all HTPs. (6) Different tobacco use definitions are considered by studies, which should be taken into account when interpreting the figure. For more information on tobacco use definitions, see Tables 1 and 2.

### Cigarette and HTP Transitions

Five studies (2 cross-sectional and 3 longitudinal) reported cigarette and HTP transitions. Tables S3 and S4 of Supplement 2 present the characteristics of these studies and the reported transition rates.

#### Cross-Sectional Studies

Two cigarette industry-sponsored studies reported cigarette and HTP transitions using a retrospective approach. Jones et al.^60^ (ages ≥20, BAT) reported that among those who exclusively smoked in 2018, 7.0% initiated using HTPs in 2019, of which 4.9% were dual and 2.1% were exclusive users. Among individuals reporting tobacco use, 5.3% of those exclusively smoking, 2.9% of those exclusively using HTPs, and 3.6% of dual-users quit both cigarettes and HTPs. Fischer et al.^59^ (ages ≥20, PMI) reported that among JGAP participants who were never tobacco users in the previous year (*n* = 3000–4000), initiation with cigarettes (7–10 participants each year) was higher than initiation with IQOS (1–5 participants each year) in 2016/17 and 2018/19, but with very small sample sizes as noted above.

#### Longitudinal-Based Studies

Applying a prospective approach, Hori, Tabuchi, and Kunugita^65^ (ages ≥15) reported transitions from never, current, and former cigarette use over 2015–2016 and 2017–2018. Over the first (second) period, transitions from never-cigarette use were 3.6% (1.2%) to exclusive cigarette, 0.2% (0.5%) to dual cigarette-HTP, and 0.2% (0.4%) to exclusive HTP. Transitions from current cigarette use included 1.9% (17.9%) to dual cigarette-HTP, 0.2% (7.6%) to exclusive HTP, and 10.9% (9.9%) to no cigarette and HTP use. Transitions from former cigarette use included 5.9% (6.5%) to exclusive cigarette, 0.2% (1.8%) to dual cigarette-HTP, and 0.5% (2.6%) to exclusive HTP use. Using a 2019 baseline and 2020 follow-up, Matsuyama and Tabuchi^68^ (ages ≥18) reported that HTP use was not associated with relapse or the initiation of cigarette use among those who recently quit cigarette use but was associated with relapse or cigarette initiation among those long-term former and never-smokers. Analyzing 2019–2021 JASTIS, Odani et al.^61^ (ages ≥20) reported that HTP use was associated with a lower likelihood of *>*1-month cigarette cessation among those who were current established (regular) cigarette users that used evidence-based cessation measures, heavy cigarette users, and less educated. A limitation of the JASTIS studies is that HTP use status at baseline is not made explicit in the reported cigarette initiation and relapse rates.

#### Summary

A BAT-sponsored study reported high rates of past-year switching from cigarettes and from dual-use to either exclusive HTP or no tobacco use in 2019.^60^ JASTIS studies reported relatively high yearly transitions from never to current cigarette smoking and from current cigarette to exclusive HTP or no HTP or cigarette use.^65^ JASTIS studies also reported that HTP use was associated with relapse or cigarette initiation among individuals who had maintained long-term (but not short-term) cigarette cessation.^68^ They also reported that HTP use was associated with a decreased likelihood of 1-month+ cigarette cessation among those who were current established (regular) cigarette users.^61^

## Discussion

Our scoping review considered a broad range of studies on HTP and cigarette use in Japan. The decline in smoking prevalence through 2018 suggests that HTP use likely contributed to a reduction in cigarette use. After 2018, the slowing sales of HTPs and studies reporting mixed results on cigarette and HTP use raise uncertainty about whether HTPs are contributing to reductions in cigarette use.

A recent review found that HTP use acts as a gateway to smoking and discourages cigarette cessation.^34^ That review, however, included studies published through 2022 and combined data from countries with different regulations on harm-reduction tobacco products (eg, e-cigarette availability differs between Japan [not legally sold] and Korea [legally sold]), which may influence the nature of HTP transitions.^21^ Although HTP use is most prevalent among those below age 30 (when cigarette initiation often takes place),^50^^,^^65^ the claim that HTPs act as a gateway to cigarette use is unclear since evidence indicates that HTPs are mainly used by current and former smokers with minimal uptake by those who have never smoked.^65^^,^^67^ Our review, on the other hand, indicates considerable heterogeneity across studies, especially regarding the different trends in HTP vis-à-vis’ cigarette use denoted by government-sponsored and online surveys. Below, we discuss issues that merit further consideration when interpreting the results of our review study.

### Differences Across Surveys

The results are not directly comparable across studies due to differences in survey structure and methodology (eg, differences in survey questions and definitions of product use, modes of survey completion, and survey dates). The current study highlights the challenges in distinguishing the impact of HTPs and cigarette use in Japan among heterogeneous surveys and studies.

The different results from government-sponsored versus online surveys may reflect differing participants. The NHNS applies a direct interview approach combined with a full-scale household-based study, which may reflect a bias toward individuals living in a family.^69^ JASTIS or other internet-based surveys, on the other hand, are often biased toward individuals living alone, who may be more likely to use HTPs while continuing to smoke.^70^^,^^71^

The differing results of government-sponsored versus online surveys may also reflect the questions asked.^50^ In particular, the interpretation of the term “smoking” and changes in questions in the government-sponsored NHNS may have influenced the findings.^50^^,^^72^ Since 2018, the NHNS has followed the question asking if the individual smokes tobacco with a sub-question asking about the use of specific products, which may underestimate cigarette use.^50^ Further, a JASTIS study^62^ found considerable denial of tobacco use relative to responses about specific product use in Japan, suggesting that respondents’ answers may reflect their view of what constitutes smoking or tobacco use. Additionally, some JASTIS studies appear to provide very different prevalence estimates for the same year despite similar questions regarding cigarette and HTP use.^17^^,^^61^

### Tobacco Use Measures

Tobacco use definitions and measures differed between surveys, with “every day or some days” used in NHNS and CSLC,^45^^,^^46^ and “past-30-day use” being the most common measures in online studies.^47^^,^^48^ Both types of measures may correspond to transitional rather than regular use, particularly as applied to dual-use.^33^^,^^73^ Measures of more regular use are relevant in gauging public health outcomes^73^ and are particularly important in new product introductions where experimental use is likely to be more common. In particular, less intensive HTP users may be less likely to quit cigarettes than more intensive users. Unlike other studies, industry-sponsored studies employed a “100-lifetime cigarette and HTP use” criterion to ensure more regular users.^58–60^^,^^74^ Frequency of cigarette and HTP use also merits consideration since more intensive HTP users may be more likely to switch completely to HTPs. For example, previous studies indicate that more intensive e-cigarette use is associated with a greater likelihood of switching from cigarettes to e-cigarettes.^75^^,^^76^ The role of dual use, in particular, may depend on how the intensity of cigarette and HTP use is measured,^77^ since current evidence does not distinguish minimal use of either product. Cigarette companies should be encouraged to make their data publicly available so that others can test the sensitivity of results to the methods adopted. At the same time, non-industry surveys should provide information to develop common measures based on patterns of regular use so that studies can more readily be compared.

**Figure 4:**
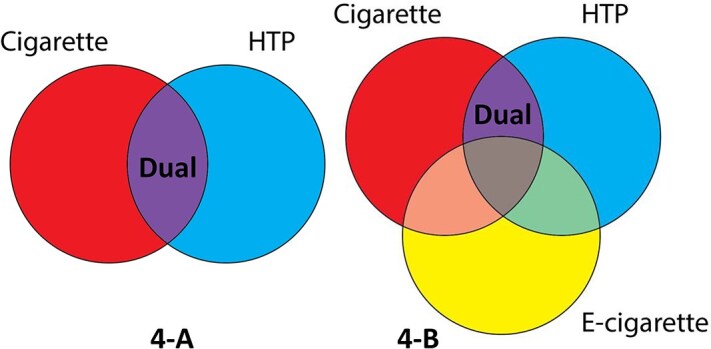
Difference between prevalence definitions among studies.

The definitions of exclusive and dual use also merit consideration. As shown in Figure 4A, most studies defined exclusive cigarette, exclusive HTP, and dual-use based only on cigarette and HTP use. More specifically, as indicated in the purple section of Figure 4A, dual cigarette-HTP users are those who used both cigarettes and HTPs, regardless of using other tobacco. However, as shown in Figure 4B, some studies (eg, Fischer et al.^59^ and Sugiyama and Tabuchi^4^) defined exclusive cigarette, exclusive HTP, and dual cigarette-HTP use, excluding participants who used other nicotine delivery products (eg, e-cigarettes). Studies excluding other tobacco use mostly reported lower exclusive cigarette, exclusive HTP, and dual cigarette-HTP prevalence.

### The Role of E-Cigarette Use

Further research is needed on the role of e-cigarette use since e-cigarettes may have been mistaken for HTPs in some surveys and, like HTPs, e-cigarette use may act as a substitute for cigarette use or may encourage the use of other nicotine delivery products.^73^^,^^78^ A JASTIS study^66^ reported that e-cigarette prevalence increased from 1.3% to 1.9% in January–February 2015–2017, and an industry study reported that e-cigarette use increased from 0.7% in 2016/17 to 2.0% in 2018/19.^59^ As indicated above and depicted in Figure 4, the definitions of cigarette and HTP use, especially regarding exclusive versus dual-use, differed between studies in terms of whether those also using other products, especially e-cigarettes, were included among the exclusive and dual HTP-cigarette use. Studies excluding e-cigarette use (eg, Fischer et al.^59^ and Sugiyama and Tabuchi^4^) mostly reported lower exclusive cigarette, exclusive HTP, and dual cigarette-HTP prevalence. Although nicotine-containing e-cigarettes are not legally sold in the domestic market of Japan, smokers who tried to quit by using HTPs and failed may have instead increasingly turned to e-cigarette use, especially since 2018.^4^^,^^79^^,^^80^ The illicit e-cigarette market in Japan is also growing, especially by importing from China.^81^ In future research, it will be important to consider the role of e-cigarette use while acknowledging that survey respondents may be reluctant to report such use due to its illegality.

### Tobacco Industry Behavior

Trends in cigarette and HTP use may also reflect industry behavior. PMI had a relatively low 23% cigarette market share in Japan before 2015, which may have encouraged their promotion of HTPs as an alternative to cigarettes sold by JTI (60% market share).^82^ Unlike Japan, competition from independent e-cigarette companies in countries where nicotine-containing e-cigarettes are legally available has played a role in encouraging those smoking to switch away from cigarettes.^73^^,^^78^^,^^83–85^ After actively competing for HTP and cigarette sales,^86^ firms may have recognized that a less competitive strategy toward cigarette use was in their joint interest.^87^ From the perspective of cigarette companies, HTPs provide high-profit margins relative to e-cigarettes.^33^ As such, the Japanese government’s complete ownership of JTI through 2003 and later partial ownership of JTI may have influenced the restrictions placed on the sale of e-cigarettes,^88–91^ thereby effectively acting as an entry barrier. Japan also lacks a public health-oriented regulatory body independent of the tobacco industry to encourage harm reduction.^92^

### Tobacco Use Policies and the COVID Pandemic

Another factor that may impact HTP and cigarette use is the changing role of cigarette- and HTP-oriented policies. Between 2018 and 2020, Japan’s MPOWER scores improved slightly in mass media, warning labels, and smoke-free protections.^42^ In 2018, Japan announced a plan to increase text-only health warnings on cigarette packs from 30% to 50%, implemented in 2019–2020. However, those changes were not found to be statistically significant.^93^ Stronger clean air laws were implemented in Japan in 2020,^94^ but the ambiguous applicability of those laws to HTPs^95^ and the ability to use HTPs in smoke-free locations may have encouraged dual-use.^96^ In addition, the cigarette and HTP tax/price increases in 2018 and 2020^97^ have been found to reduce cigarette initiation and relapse and increase cigarette cessation,^97–99^ but HTPs were less taxed in terms of tax-based units.^100^ The role of COVID also merits attention. A study found that exclusive HTP use was associated with quitting tobacco during the pandemic, while dual-users increased cigarette use intensity.^101^

### Other Limitations

In addition to not explicitly considering the role of tobacco control policies, we focused on pre-HTP cigarette use trends from 2010 to 2014 but did not explicitly estimate and distinguish more long-term declines in cigarette use. While those factors may have played an important role and merit further consideration, attempts to distinguish their role would have added considerable complexity to our analysis. Another limitation is that we did not conduct a single quality assessment to evaluate the limitations of each study, due to the heterogeneity of studies and the diversity of their methods and aims. Instead, we informally appraised the quality of the reviewed studies and reported any identified issues. Some examples of such limitations noted during our review include: (1) frequent changes made to the NHNS questions,^50^ (2) less intensive tobacco use measure used in most studies, which may correspond to transitional rather than regular use,^33^^,^^73^ (3) excluding “other tobacco use” from exclusive and dual use definitions in some studies,^4^^,^^59^ (4) several JASTIS studies for the same year provided very different prevalence estimates despite similar questions regarding cigarette and HTP use,^17^^,^^61^ and (5) not reporting HTP use status in some studies that reported cigarette initiation and relapse rates.^61^^,^^68^ As another potential source of bias, we distinguished tobacco industry-funded from other studies. Lastly, we limited our search to studies published in English. Studies published in Japanese may have been overlooked.

## Conclusions

Over the past decade, Japan has been a testing ground for marketing HTPs. Our scoping review obtains consistent findings of HTP use likely contributing to a decrease in cigarette use from 2014 to 2018 following their introduction into Japan. However, government-sponsored and online surveys indicate different impacts of HTP use on exclusive versus dual cigarette use after 2018. Consequently, the evidence remains incomplete, limiting definitive conclusions; further research is needed to support any causal relationship between HTP and cigarette use. Our study highlights challenges in distinguishing the impact of HTPs in an environment subject to rapidly evolving tobacco products, patterns of use, tobacco control policies, and other dynamic factors. Further research is needed, especially regarding the role of e-cigarettes, the role of tobacco control policies, and the variation in results from government-sponsored and online surveys.

## Supplementary Material

Japan_cigarette_and_HTP_review_Supplements-Final-08-14-25_ntaf216

## Data Availability

Data are available upon reasonable request to the corresponding author.
