# Supplementary material for: Recent Trends in Cigarette and HTP Use in Japan: A Scoping Review
Source: Nicotine Tob Res. 2025 Oct 24;28(4):511–25. doi: 10.1093/ntr/ntaf216 (PMC13008579; doi:10.1093/ntr/ntaf216)
Supplement: Japan_cigarette_and_HTP_review_Supplements-Final-08-14-25_ntaf216 [file japan_cigarette_and_htp_review_supplements-final-08-14-25_ntaf216.pdf]

Supplement 1

The complete search strategy for the PubMed database

(("japan*"[Title/Abstract]) AND ("heat* tobacco"[All Fields] OR "HTP"[All Fields] OR "heat-not-burn"[All Fields] OR "heat not burn"[All Fields] OR "IQOS"[All Fields] OR "glo"[All Fields] OR "Ploom"[All Fields] OR "tobacco products"[MeSH Terms] OR "smoking"[MeSH Terms])) AND ("trend"[Title/Abstract] OR "transition"[Title/Abstract] OR "prevalence"[Title/Abstract] OR "pattern"[Title/Abstract] OR "chang*"[Title/Abstract] OR "shift"[Title/Abstract] OR "evolution"[Title/Abstract] OR "sale"[Title/Abstract] OR "switch*"[Title/Abstract] OR "initiat*"[Title/Abstract] OR "cessation"[Title/Abstract] OR "quit*"[Title/Abstract] OR "use"[Title/Abstract]) AND (2010:2024[pdat]).

Supplement 1-Table S1: Exclusion reasons for articles that received a full-text review.

Screened Article	Exclusion Reason
Kanai M, Kanai O, Tabuchi T, Mio T. Association of heated tobacco product use with tobacco use cessation in a Japanese workplace: a prospective study. Thorax. 2021;76(6):615-617.	A clinical trial with N=158 participants
Myagmar-Ochir E, .: a cross-sectional analysis of retail workers in Japan. BMJ open. 2021;11(8):e049395.	Sub-population: Only focused on occupational differences in using HTPs
Yamada K, Imano H, Tabuchi T, et al. Longitudinal trajectories of smoking status using 25 year annually-updated data and all-cause mortality followed over 30 years: A community-based prospective cohort study. Preventive Medicine. 2023:107575.	Smoking status is reported for 2009 and before.
Tattan-Birch H, Hartmann-Boyce J, Kock L, et al. Heated tobacco products for smoking cessation and reducing smoking prevalence. Cochrane Database of Systematic Reviews. 2022(1).	This review paper included 11 randomized clinical trials and 2 time series. We excluded clinical trials and have discussed the two time series in our study.
Katanoda K, Jiang Y, Park S, Lim MK, Qiao Y-L, Inoue M. Tobacco control challenges in East Asia: proposals for change in the world's largest epidemic region. Tobacco Control. 2014;23(4):359-368.	The 2010 smoking prevalence from NHNS is reported (already included).
Tabuchi T, Kondo N. Educational inequalities in smoking among Japanese adults aged 25–94 years: Nationally representative sex-and age-specific statistics. Journal of epidemiology. 2017;27(4):186-192.	The 2010 smoking prevalence from CSLC (divided by educational attainment) is reported (already included).
Tabuchi T, Hoshino T, Hama H, et al. Complete workplace indoor smoking ban and smoking behavior among male workers and female nonsmoking workers' husbands: A pseudo cohort study of Japanese public workers. BioMed Research International. 2014;2014.	The 2010 smoking prevalence from CSLC is reported.

Tabuchi T, Nakamura M, Nakayama T, Miyashiro I, Mori J-i, Tsukuma H. Tobacco price increase and smoking cessation in Japan, a developed country with affordable tobacco: a national population-based observational study. Journal of epidemiology. 2016;26(1):14-21.	The 2010 smoking prevalence from NHNS and CSLC is reported.
Tomioka K, Kurumatani N, Saeki K. The association between education and smoking prevalence, independent of occupation: a nationally representative survey in Japan. Journal of epidemiology. 2020;30(3):136-142.	The 2010 smoking prevalence from CSLC is reported.
Osaki Y, Maesato H, Minobe R, et al. Changes in smoking behavior among victims after the great East Japan earthquake and tsunami. Environmental Health and Preventive Medicine. 2020;25(1):1-8.	No outcome of interest
Nakano H, Ohira T, Maeda M, et al. Associations of disaster-related and psychosocial factors with changes in smoking status after a disaster: a cross-sectional survey after the Great East Japan Earthquake. BMJ open. 2018;8(6):e018943.	No outcome of interest
Xu SS, Meng G, Yan M, et al. Reasons for regularly using heated tobacco products among adult current and former smokers in Japan: finding from 2018 ITC Japan Survey. International Journal of Environmental Research and Public Health. 2020;17(21):8030.	No outcome of interest
Lau YK, Okawa S, Meza R, Katanoda K, Tabuchi T. Nicotine dependence of cigarette and heated tobacco users in Japan, 2019: a cross-sectional analysis of the JASTIS study. Tobacco Control. 2022;31(e1):e50-e56.	No outcome of interest
Igarashi A, Negishi S, Goto R, Suwa K. Web-based survey on smoking cessation behaviors of current and former smokers in Japan. Current Medical Research and Opinion. 2014;30(10):1911-1921.	No outcome of interest
Tabuchi T, Hoshino T, Nakayama T. Are partial workplace smoking bans as effective as complete smoking bans? A national population-based study of smoke-free policy among Japanese employees. Nicotine & Tobacco Research. 2016;18(5):1265-1273.	No outcome of interest
Kiyohara K, Tabuchi T. Use of heated tobacco products in smoke-free locations in Japan: the JASTIS 2019 study. Tob Control. 2020.	No outcome of interest
Siripongvutikorn Y, Tabuchi T, Okawa S. Workplace smoke-free policies that allow heated tobacco products and electronic cigarettes use are associated with use of both these products and conventional tobacco smoking: the 2018 JASTIS study. Tobacco Control. 2021;30(2):147-154.	No outcome of interest
Tanihara S, Momose Y. Reasons for smoking cessation attempts among Japanese male smokers vary by nicotine dependence level: a cross-sectional study after the 2010 tobacco tax increase. BMJ open. 2015;5(3):e006658.	No outcome of interest
Craig LV, Yoshimi I, Fong GT, et al. Awareness of marketing of heated tobacco products and cigarettes and support for tobacco marketing restrictions in Japan: findings from the 2018 international tobacco control (ITC) Japan survey. International Journal of Environmental Research and Public Health. 2020;17(22):8418.	No outcome of interest

Sutanto E, Smith DM, Miller C, et al. Use of heated tobacco products within indoor spaces: findings from the 2018 ITC Japan survey. International Journal of Environmental Research and Public Health. 2019;16(23):4862.	No outcome of interest<br>The 2018 prevalence data from ITC is already included in the results.
Tabuchi T, Kiyohara K, Hoshino T, Bekki K, Inaba Y, Kunugita N. Awareness and use of electronic cigarettes and heat-not-burn tobacco products in Japan. Addiction. 2016;111(4):706-713.	They reported the prevalence of e-cigarettes and HTPs (together). Additionally, the 2015 JASTIS prevalence is already included in the results.
Koyama S, Tabuchi T, Okawa S, et al. Changes in smoking behavior since the declaration of the COVID-19 state of emergency in Japan: a cross-sectional study from the Osaka health app.	No outcome of interest. This study focuses on the effect of COVID on smoking behavior change.
Matsubayashi K, Tabuchi T, Iso H. Tobacco price increase and successful smoking cessation for two or more years in Japan. Nicotine and Tobacco Research. 2021;23(4):716-723.	No outcome of interest. This study focuses on the effect of tobacco price increase on smoking cessation during 2005-2011.
Tabuchi T, Fujiwara T, Shinozaki T. Tobacco price increase and smoking behaviour changes in various subgroups: a nationwide longitudinal 7-year follow-up study among a middle-aged Japanese population. Tobacco control. 2017;26(1):69-77.	No outcome of interest. This study focuses on the effect of tobacco price increase on smoking cessation during 2005-2011.
Kanai M, Kanai O, Tabuchi T. Impact of the COVID-19 pandemic on changes in tobacco use behaviour: A longitudinal cohort study in Japan. Journal of Epidemiology. 2024:JE20240180.	Transitions reported for conventional tobacco (cigarette, hand-rolled tobacco, cigar, little cigar, pipe tobacco), and novel tobacco (HTPs, e-cigarettes). Also, focused on the effect of COVID.
Yamamoto T, Abbas H, Kanai M, Yokoyama T, Tabuchi T. Factors associated with smoking behaviour changes during the COVID-19 pandemic in Japan: a 6-month follow-up study. Tobacco Control. 2022	A small cohort from JASTIS is considered to evaluate the effect of COVID pandemic
Togawa K, Fong GT, Quah AC, et al. Impacts of revised smoke-free regulations under the 2020 Japan Health Promotion Act on cigarette smoking and heated tobacco product use in indoor public places and homes: findings from 2018 to 2021 International Tobacco Control (ITC) Japan Surveys. Tobacco Control. 2024.	Prevalence of cigarettes and HTPs in indoor spaces is discussed
Matsuyama Y, Tabuchi T. Stepwise Tobacco Price Increase and Smoking Behavioral Changes in Japan: The Japan "Society and New Tobacco" Internet Survey 2017–2021 Longitudinal Study. Nicotine & Tobacco Research. 2022.	The impact of tobacco price increase on cigarette transitions is studied
Harada S, Sata M, Matsumoto M, et al. Changes in Smoking Habits and Behaviors Following the Introduction and Spread of Heated Tobacco Products in Japan and Its Effect on FEV1 Decline: A Longitudinal Cohort Study. Journal of epidemiology. 2022;32(4):180-187.	Focused on health effects of HTPs

Sansone G, Fong GT, Meng G, et al. Secondhand smoke exposure in public places and support for smoke-free laws in Japan: Findings from the 2018 ITC Japan Survey. International Journal of Environmental Research and Public Health. 2020;17(3):979.	Focused on second-hand smoke
Adamson J, Kanitscheider C, Prasad K, et al. Results from a 2018 cross-sectional survey in Tokyo, Osaka and Sendai to assess tobacco and nicotine product usage after the introduction of heated tobacco products (HTPs) in Japan. Harm reduction journal. 2020;17(1):1-10.	Non nationally representative
garashi A, Aida J, Kusama T, et al. Heated Tobacco Products Have Reached Younger or More Affluent People in Japan. J Epidemiol. Mar 28 2020;doi:10.2188/jea.JE20190260	Non nationally representative

Supplement 2

Supplement 2-Table S1: Cigarette and dual prevalence reported by the reviewed papers.

Paper	Prevalence type	Tobacco Use definition	Age range	Sex	Year													
					2010	2011	2012	2013	2014	2015	2016	2017	2018	2019	2020	2021	2022	2023
Okui[50] (NHNS)	Age-adjusted total cigarette prevalence	Habitually smoking	20+	Men	37.7	38.3	39.7	39	39.1	35.5	36.6	34.1	33.2	-	-	-	-	-
				Women	11.4	12.8	11.5	11.3	11.7	9.8	10.7	9	10.6	-	-	-	-	-
Sugiyama and Tabuchi[4]* (JASTIS)	Exclusive cigarette prevalence	Past 30-days use	15+	Overall	-	-	-	-	-	-	-	17	-	-	-	-	-	-
			15–24		-	-	-	-	-	-	-	6.6	-	-	-	-	-	-
			25–34		-	-	-	-	-	-	-	14.6	-	-	-	-	-	-
			35–44		-	-	-	-	-	-	-	19.3	-	-	-	-	-	-
			45–54		-	-	-	-	-	-	-	20.5	-	-	-	-	-	-
			55–64		-	-	-	-	-	-	-	20.7	-	-	-	-	-	-
			65–71		-	-	-	-	-	-	-	14.1	-	-	-	-	-	-
			15+	Men	-	-	-	-	-	-	-	26.3	-	-	-	-	-	-
			15+	Women	-	-	-	-	-	-	-	7.4	-	-	-	-	-	-
	Dual cigarette-HTP prevalence	Past 30-day use	15+	Overall	-	-	-	-	-	-	-	1.6	-	-	-	-	-	-
Odani and Tabuchi[17] (JASTIS)	Total cigarette prevalence	Past 30-days use	15+	Overall	-	-	-	-	-	-	-	-	-	-	25.9	-	-	-
	Dual cigarette-HTP prevalence	Past 30-day use	15+	Overall	-	-	-	-	-	-	-	-	-	-	8.6	-	-	-
			15-19		-	-	-	-	-	-	-	-	-	-	4	-	-	-
			20-29		-	-	-	-	-	-	-	-	-	-	15.1	-	-	-
			30-39		-	-	-	-	-	-	-	-	-	-	13.6	-	-	-
			40-49		-	-	-	-	-	-	-	-	-	-	9.9	-	-	-
			50-59		-	-	-	-	-	-	-	-	-	-	8.1	-	-	-
			60+		-	-	-	-	-	-	-	-	-	-	4.1	-	-	-
			15+	Men	-	-	-	-	-	-	-	-	-	-	13.5	-	-	-
			15+	Women	-	-	-	-	-	-	-	-	-	-	4.4	-	-	-
Odani and Tabuchi[63] (JASTIS)	Total cigarette prevalence	Past 30-days use	16+	Overall	-	-	-	-	-	-	-	-	-	-	-	-	19.4	-
			16-19		-	-	-	-	-	-	-	-	-	-	-	-	4.2	-
			20-29		-	-	-	-	-	-	-	-	-	-	-	-	20.1	-
			30-39		-	-	-	-	-	-	-	-	-	-	-	-	23.1	-

			40-49		-	-	-	-	-	-	-	-	-	-	-	-	22.6	-
			50-59		-	-	-	-	-	-	-	-	-	-	-	-	22.4	-
			60-74		-	-	-	-	-	-	-	-	-	-	-	-	16	-
			16+	Men	-	-	-	-	-	-	-	-	-	-	-	-	28.7	-
			16+	Women	-	-	-	-	-	-	-	-	-	-	-	-	10.5	-
Yamamoto et al.[65]*	Exclusive cigarette prevalence	Past 30-days use	≥17	Overall	-	-	-	-	-	-	-	-	-	-	-	-	8.7	-
	Dual cigarette-HTP prevalence	Past 30-day use	≥17	Overall	-	-	-	-	-	-	-	-	-	-	-	-	3.2	-
Odani and Tabuchi[64]	Total cigarette prevalence	Past 30-days use	≥16	Overall	-	-	-	-	-	-	-	-	-	-	-	-	-	18.9
			16-19		-	-	-	-	-	-	-	-	-	-	-	-	-	6.8
			20-29		-	-	-	-	-	-	-	-	-	-	-	-	-	16.8
			30-39		-	-	-	-	-	-	-	-	-	-	-	-	-	18.7
			40-49		-	-	-	-	-	-	-	-	-	-	-	-	-	23.4
			50-59		-	-	-	-	-	-	-	-	-	-	-	-	-	20.6
			60-74		-	-	-	-	-	-	-	-	-	-	-	-	-	17.2
			≥16	Men	-	-	-	-	-	-	-	-	-	-	-	-	-	27.6
			16-19		-	-	-	-	-	-	-	-	-	-	-	-	-	9.9
			20-29		-	-	-	-	-	-	-	-	-	-	-	-	-	25.0
			30-39		-	-	-	-	-	-	-	-	-	-	-	-	-	28.2
			40-49		-	-	-	-	-	-	-	-	-	-	-	-	-	32.0
			50-59		-	-	-	-	-	-	-	-	-	-	-	-	-	30.1
			60-74		-	-	-	-	-	-	-	-	-	-	-	-	-	25.8
			≥16	Women	-	-	-	-	-	-	-	-	-	-	-	-	-	10.4
			16-19		-	-	-	-	-	-	-	-	-	-	-	-	-	4.4
			20-29		-	-	-	-	-	-	-	-	-	-	-	-	-	9.0
			30-39		-	-	-	-	-	-	-	-	-	-	-	-	-	9.7
			40-49		-	-	-	-	-	-	-	-	-	-	-	-	-	14.7
			50-59		-	-	-	-	-	-	-	-	-	-	-	-	-	11.3
			60-74		-	-	-	-	-	-	-	-	-	-	-	-	-	8.8
	Dual combustible-HTP prevalence	Past 30-days use	≥16	Overall	-	-	-	-	-	-	-	-	-	-	-	-	-	7.4
			16-19		-	-	-	-	-	-	-	-	-	-	-	-	-	5.5
			20-29		-	-	-	-	-	-	-	-	-	-	-	-	-	11.1
			30-39		-	-	-	-	-	-	-	-	-	-	-	-	-	9.4

			40-49		-	-	-	-	-	-	-	-	-	-	-	-	-	8.8
			50-59		-	-	-	-	-	-	-	-	-	-	-	-	-	5.5
			60-74		-	-	-	-	-	-	-	-	-	-	-	-	-	3.4
			≥16	Men	-	-	-	-	-	-	-	-	-	-	-	-	-	11.6
			16-19	Men	-	-	-	-	-	-	-	-	-	-	-	-	-	8.3
			20-29	Men	-	-	-	-	-	-	-	-	-	-	-	-	-	16.8
			30-39	Men	-	-	-	-	-	-	-	-	-	-	-	-	-	15.2
			40-49	Men	-	-	-	-	-	-	-	-	-	-	-	-	-	13.2
			50-59	Men	-	-	-	-	-	-	-	-	-	-	-	-	-	8.5
			60-74	Men	-	-	-	-	-	-	-	-	-	-	-	-	-	5.7
			≥16	Women	-	-	-	-	-	-	-	-	-	-	-	-	-	3.4
			16-19	Women	-	-	-	-	-	-	-	-	-	-	-	-	-	3.4
			20-29	Women	-	-	-	-	-	-	-	-	-	-	-	-	-	5.6
			30-39	Women	-	-	-	-	-	-	-	-	-	-	-	-	-	3.9
			40-49	Women	-	-	-	-	-	-	-	-	-	-	-	-	-	4.4
			50-59	Women	-	-	-	-	-	-	-	-	-	-	-	-	-	2.6
			60-74	Women	-	-	-	-	-	-	-	-	-	-	-	-	-	1.1
Odani[62] (JASTIS)	Total cigarette prevalence	Past 30-days use	20+	Overall	-	-	-	-	-	-	-	-	-	16.4	18.6	-	-	-
			20-29	Overall	-	-	-	-	-	-	-	-	-	12.4	13.6	-	-	-
			30-39	Overall	-	-	-	-	-	-	-	-	-	14.7	19.5	-	-	-
			40-49	Overall	-	-	-	-	-	-	-	-	-	19.1	21.7	-	-	-
			50-59	Overall	-	-	-	-	-	-	-	-	-	20.4	21.1	-	-	-
			60-74	Overall	-	-	-	-	-	-	-	-	-	14.3	16	-	-	-
			20+	Men	-	-	-	-	-	-	-	-	-	23.1	24.6	-	-	-
			20+	Women	-	-	-	-	-	-	-	-	-	8.7	10.4	-	-	-
	Dual cigarette-HTP prevalence	Past 30-days use	20+	Overall	-	-	-	-	-	-	-	-	-	6.4	5.9	-	-	-
			20-29	Overall	-	-	-	-	-	-	-	-	-	7	8.3	-	-	-
			30-39	Overall	-	-	-	-	-	-	-	-	-	7.6	7.8	-	-	-
			40-49	Overall	-	-	-	-	-	-	-	-	-	7.8	6.1	-	-	-
			50-59	Overall	-	-	-	-	-	-	-	-	-	6.5	6.2	-	-	-
			60-74	Overall	-	-	-	-	-	-	-	-	-	4.2	2.9	-	-	-
			20+	Men	-	-	-	-	-	-	-	-	-	9.5	8.3	-	-	-
			20+	Women	-	-	-	-	-	-	-	-	-	2.8	3.2	-	-	-
Kuwabara et al.[56,57] (LSA)	Total cigarette prevalence	Past 30-days use	13-15	Overall	-	-	-	-	-	-	-	0.6	-	-	-	-	-	-
			13-15	Men	-	-	-	-	-	-	-	0.7	-	-	-	-	-	-
			13-15	Women	-	-	-	-	-	-	-	0.5	-	-	-	-	-	-

			16-18	Overall	-	-	-	-	-	-	-	1.5	-	-	-	-	-	-
				Men	-	-	-	-	-	-	-	2	-	-	-	-	-	-
				Women	-	-	-	-	-	-	-	0.9	-	-	-	-	-	-
Sutanto[68] (ITC)	Dual cigarette-HTP prevalence	Past 30-day use	20+	Overall	-	-	-	-	-	-	-	-	1.8	-	-	-	-	-
Kinjo et al.[2] (PBSHTS)	Exclusive combustible tobacco prevalence	Past 30-days use	20+	Overall	-	-	-	-	-	-	-	-	14.5	-	-	-	-	-
				Men	-	-	-	-	-	-	-	-	22.3	-	-	-	-	-
				Women	-	-	-	-	-	-	-	-	7.9	-	-	-	-	-
Fischer[60] (PMI/GJAP)	Total cigarette prevalence	Everyday or someday use with 100 cigarettes use lifetime	20+	Overall	-	-	-	-	-	-	-	17.6	17.3	16	-	-	-	-
			20-29		-	-	-	-	-	-	-	18.6	15.1	15.4	-	-	-	-
			30-39		-	-	-	-	-	-	-	23.9	21.8	20.2	-	-	-	-
			40-49		-	-	-	-	-	-	-	24.6	22.2	20.4	-	-	-	-
			50+		-	-	-	-	-	-	-	13.5	15	13.8	-	-	-	-
			20+	Men	-	-	-	-	-	-	-	28.2	28.4	26.3	-	-	-	-
			20+	Women	-	-	-	-	-	-	-	7.9	7.7	7	-	-	-	-
Jones et al.[61]	Total cigarette prevalence	At least 100 times use lifetime	≥20	Overall	-	-	-	-	-	-	-	-	-	14.6	-	-	-	-

Notes:

Bolded Italicized studies are Industry-sponsored.

*Exclusive cigarette prevalence means “only cigarette use and no other tobacco use,” and dual cigarette-HTP prevalence mean “only dual cigarette-HTP use and no other tobacco use”

Different tobacco use definitions are considered by studies, which should be taken into account when interpreting the table. For more information on tobacco use definitions see Tables 1&2.

Supplement 2-Table S2: HTP prevalence reported by the reviewed papers.

Paper	Prevalence type	Tobacco Use definition	Age range	Sex	Year								
					2015	2016	2017	2018	2019	2020	2021	2022	2023
Kinjo[2]	Total HTP prevalence crude rate	Past 30 day use	20+	Men	-	-	-	7.3	-	-	-	-	-
			20–29		-	-	-	12.6	-	-	-	-	-
			30–39		-	-	-	13.4	-	-	-	-	-
			40–49		-	-	-	13.3	-	-	-	-	-
			50–59		-	-	-	7.7	-	-	-	-	-
			60–69		-	-	-	5.6	-	-	-	-	-
			70–79		-	-	-	0.7	-	-	-	-	-
			80+		-	-	-	0	-	-	-	-	-
			20+	Women	-	-	-	1.8	-	-	-	-	-
			20–29		-	-	-	4.1	-	-	-	-	-
			30–39		-	-	-	4.7	-	-	-	-	-
			40–49		-	-	-	2.5	-	-	-	-	-
			50–59		-	-	-	2.3	-	-	-	-	-
			60–69		-	-	-	0.2	-	-	-	-	-
			70–79		-	-	-	0	-	-	-	-	-
			80+		-	-	-	0	-	-	-	-	-
Sugiyama and Tabuchi*[4] (JASTIS)	Exclusive HTP prevalence	Past 30-days use	15+	Overall	-	-	1.1	-	-	-	-	-	-
Hori, Tabuchi, Kunugita[15] (JASTIS)	Total HTP prevalence	Past 30-day use	15+	Overall	0.2	0.8	3.7	8	11.3	-	-	-	-
			15-19		0.6	2	1.4	7.4	4.8	-	-	-	-
			20-29		0.2	1	5.7	14.1	17	-	-	-	-
			30-39		0.3	1.2	5.1	9	15.2	-	-	-	-
			40-49		0	0.4	4	8.5	12.5	-	-	-	-
			50-59		0	0.5	3.5	6.8	9.1	-	-	-	-
			60-69		0	0.1	0.6	2	3.3	-	-	-	-
			15+	Men	0.3	1.2	5.6	12.3	17.2	-	-	-	-
			15+	Women	0	0.3	1.8	3.9	5.6	-	-	-	-

Odani and Tabuchi[17] (JASTIS)	Total HTP prevalence	Past 30-day use	15+	Overall	-	-	-	-	-	10.9	-	-	-
			15-19		-	-	-	-	-	4.3	-	-	-
			20-29		-	-	-	-	-	15.9	-	-	-
			30-39		-	-	-	-	-	16.9	-	-	-
			40-49		-	-	-	-	-	14.8	-	-	-
			50-59		-	-	-	-	-	9.9	-	-	-
			60+		-	-	-	-	-	4.2	-	-	-
			15+	Men	-	-	-	-	-	16.6	-	-	-
			15+	Women	-	-	-	-	-	5.5	-	-	-
Odani and Tabuchi[63] (JASTIS)	Total HTP prevalence	Past 30-day use	16+	Overall	-	-	-	-	-	-	-	11.8	-
			16-19		-	-	-	-	-	-	-	3.8	-
			20-29		-	-	-	-	-	-	-	16	-
			30-39		-	-	-	-	-	-	-	17.7	-
			40-49		-	-	-	-	-	-	-	14.8	-
			50-59		-	-	-	-	-	-	-	11.6	-
			60-74		-	-	-	-	-	-	-	5.9	-
			16+	Men	-	-	-	-	-	-	-	17.9	-
			16+	Women	-	-	-	-	-	-	-	6	-
Yamamoto et al.*[65]	Exclusive HTP prevalence	Past 30-day use	≥17	Overall	-	-	-	-	-	-	-	4.7	-
Odani and Tabuchi[64]	Total HTP prevalence	Past 30-day use	≥16	Overall	-	-	-	-	-	-	-	-	12.4
			16-19		-	-	-	-	-	-	-	-	7
			20-29		-	-	-	-	-	-	-	-	14.7
			30-39		-	-	-	-	-	-	-	-	16.3
			40-49		-	-	-	-	-	-	-	-	16.4
			50-59		-	-	-	-	-	-	-	-	10.7
			60-74		-	-	-	-	-	-	-	-	6.2
			≥16	Men	-	-	-	-	-	-	-	-	18.9
			16-19		-	-	-	-	-	-	-	-	10.6
			20-29		-	-	-	-	-	-	-	-	21.6
			30-39		-	-	-	-	-	-	-	-	25.3
			40-49		-	-	-	-	-	-	-	-	24.4
			50-59		-	-	-	-	-	-	-	-	16.4
			60-74		-	-	-	-	-	-	-	-	10.2
			≥16	Women	-	-	-	-	-	-	-	-	6.1

			16-19		-	-	-	-	-	-	-	-	4.3
			20-29		-	-	-	-	-	-	-	-	8.1
			30-39		-	-	-	-	-	-	-	-	7.6
			40-49		-	-	-	-	-	-	-	-	8.4
			50-59		-	-	-	-	-	-	-	-	5.0
			60-74		-	-	-	-	-	-	-	-	2.3
Odani[62] (JASTIS)	Total HTP prevalence	Past 30-day use	20+	Overall	-	-	-	-	9.5	8.5	-	-	-
			20-29		-	-	-	-	10.3	10.1	-	-	-
			30-39		-	-	-	-	12.8	11.1	-	-	-
			40-49		-	-	-	-	11.4	10.1	-	-	-
			50-59		-	-	-	-	9.9	9.4	-	-	-
			60-74		-	-	-	-	5.3	4	-	-	-
			20+	Men	-	-	-	-	13.8	11.9	-	-	-
			20+	Women	-	-	-	-	4.7	4.5	-	-	-
Sutanto[68] (ITC)	Total HTP prevalence	Past 30-day use	20+	Overall	-	-	-	2.7	-	-	-	-	-
	Exclusive HTP prevalence	Past 30-day use	20+	Overall	-	-	-	0.9	-	-	-	-	-
Kuwabara et al.[56,57] (LSA)	Total HTP prevalence	Past 30-day use	13-15	Overall	-	-	0.5	-	-	-	-	-	-
				Men	-	-	0.6	-	-	-	-	-	-
				Women	-	-	0.4	-	-	-	-	-	-
			16-18	Overall	-	-	0.9	-	-	-	-	-	-
				Men	-	-	1.2	-	-	-	-	-	-
				Women	-	-	0.6	-	-	-	-	-	-
Jones et al.[61]	Total HTP prevalence	At least 100 times use lifetime	≥20	Overall	-	-	-	-	5.3%	-	-	-	-

Notes: ***Bolded Italicized*** studies are Industry-sponsored.
*Exclusive HTP prevalence means "only HTP use and no other tobacco use"
Different tobacco use definitions are considered by studies, which should be taken into account when interpreting the table. For more information on tobacco use definitions see Tables 1&2.

Supplement 2-Table S3: Properties of the studies that reported cigarette and HTP transitions.

Study	Data source	Nationally representative?	Cross-sectional or longitudinal?	Survey type	Years conducted	Sample size	Age	Current tobacco use definition
Jones et al.[61]	***BAT***	Yes	Cross-sectional (retrospective)	In-person	2019	5,306	≥20	At least 100 times use lifetime
Fischer et al.[60]	***PMI/ JGAP***	Yes	Cross-sectional (three periods)	In-person	2016-19	5,000	≥20	Daily or non-daily use, with at least 100 times use lifetime
Hori, Tabuchi, and Kunugita[66]	JASTIS	Yes	Longitudinal and prospective (two periods)	Online	2015-16 and 2017-18	5,366 and 3,422	≥15	past-30-day
Matsuyama and Tabuchi[69]	JASTIS	Yes	Longitudinal and prospective (2019 baseline, 2020 follow-up)	Online	2019-2020	7,766 and 5,946	≥18	past-30-day
Odani et al.[62]	JASTIS	Yes	Longitudinal and prospective (three time points, 2019 baseline, 2020 follow-up, and 2020 baseline, 2021 follow-up)	Online	2019-21	7,044	≥20	past-30-day

Note: ***Bolded Italicized*** studies are Industry-sponsored.

Supplement 2-Table S4: Cigarette and HTP transition rates.

From	To	Gender	Age	Year	Rate	Source	Note
**Never cigarette users**	**Cigarette users**	Both	18-73	2019-20	1.00%	Matsuyama and Tabuchi[69]	from never cigarette users (HTP unknown) to current cigarette users (HTP unknown)
			15+	2017-18	1.20%	Hori, Tabuchi, and Kunugita[66]	from never cigarette users (HTP unknown) to exclusive cigarette users
				2015-16	3.60%	Hori, Tabuchi, and Kunugita[66]	from never cigarette users (HTP unknown) to exclusive cigarette users
	**Dual users**		15+	2017-18	0.50%	Hori, Tabuchi, and Kunugita[66]	from never cigarette users (HTP unknown) to dual users
				2015-16	0.20%	Hori, Tabuchi, and Kunugita[66]	from never cigarette users (HTP unknown) to dual users
	**HTP users**		15+	2017-18	0.40%	Hori, Tabuchi, and Kunugita[66]	from never cigarette users (HTP unknown) to exclusive HTP users
				2015-16	0.20%	Hori, Tabuchi, and Kunugita[66]	from never cigarette users (HTP unknown) to exclusive HTP users

	**No use**		15+	2017-18	98.00%	Hori, Tabuchi, and Kunugita[66]	from never cigarette users (HTP unknown) to no use (may include former cigarette users)
				2015-16	96.00%	Hori, Tabuchi, and Kunugita[66]	from never cigarette users (HTP unknown) to no use (may include former cigarette users)
**Current Cigarette users**	**Cigarette users**	Both	20+	2018-19	82.70%	Jones et al.[61]	from exclusive cigarette users to exclusive cigarette users
			15+	2017-18	66.20%	Hori, Tabuchi, and Kunugita[66]	from current cigarette users (HTP unknown) to exclusive cigarette users
				2015-16	87.00%	Hori, Tabuchi, and Kunugita[66]	from current cigarette users (HTP unknown) to exclusive cigarette users
	**Dual Users**	Both	20+	2018-19	4.90%	Jones et al.[61]	from exclusive cigarette users to dual users
			15+	2017-18	17.90%	Hori, Tabuchi, and Kunugita[66]	from current cigarette users (HTP unknown) to dual users
				2015-16	1.90%	Hori, Tabuchi, and Kunugita[66]	from current cigarette users (HTP unknown) to dual users
	**HTP users**	Both	20+	2018-19	2.10%	Jones et al.[61]	from exclusive cigarette users to exclusive HTP users
			15+	2017-18	7.60%	Hori, Tabuchi, and Kunugita[66]	from current cigarette users (HTP unknown) to exclusive HTP users
				2015-16	0.20%	Hori, Tabuchi, and Kunugita[66]	from current cigarette users (HTP unknown) to exclusive HTP users
	**No use**	Both	20+	2018-19	5.30%	Jones et al.[61]	from exclusive cigarette users to no use (no cig and no HTP)
			15+	2017-18	9.90%	Hori, Tabuchi, and Kunugita[66]	from current cigarette users (HTP unknown) to no use (no cig and no HTP)
				2015-16	10.90%	Hori, Tabuchi, and Kunugita[66]	from current cigarette users (HTP unknown) to no use (no cig and no HTP)
			20-74	2019-21	11.30%	Odani et al.[62]	from exclusive cigarette users to ≥6mo cigarette quitter (HTP unknown)
					22.00%	Odani et al.[62]	from exclusive cigarette users to ≥1mo cigarette quitter (HTP unknown)
				2020-21	14.30%	Odani et al.[62]	from exclusive cigarette users to ≥6mo cigarette quitter (HTP unknown)
					26.60%	Odani et al.[62]	from exclusive cigarette users to ≥1mo cigarette quitter (HTP unknown)
				2019-20	8.60%	Odani et al.[62]	from exclusive cigarette users to ≥6mo cigarette quitter (HTP unknown)

					18.00%	Odani et al.[62]	from exclusive cigarette users to ≥1mo cigarette quitter (HTP unknown)
			20-29	2019-21	12.60%	Odani et al.[62]	from current cigarette users (HTP unknown) to ≥6mo cigarette quitter (HTP unknown)
					33.50%	Odani et al.[62]	from current cigarette users (HTP unknown) to ≥1mo cigarette quitter (HTP unknown)
					15.70%	Odani et al.[62]	from exclusive cigarette users to ≥6mo cigarette quitter (HTP unknown)
					38.50%	Odani et al.[62]	from exclusive cigarette users to ≥1mo cigarette quitter (HTP unknown)
			30-39	2019-21	9.30%	Odani et al.[62]	from current cigarette users (HTP unknown) to ≥6mo cigarette quitter (HTP unknown)
					20.90%	Odani et al.[62]	from current cigarette users (HTP unknown) to ≥1mo cigarette quitter (HTP unknown)
					10.00%	Odani et al.[62]	from exclusive cigarette users to ≥6mo cigarette quitter (HTP unknown)
					21.80%	Odani et al.[62]	from exclusive cigarette users to ≥1mo cigarette quitter (HTP unknown)
			40-49	2019-21	9.10%	Odani et al.[62]	from current cigarette users (HTP unknown) to ≥6mo cigarette quitter (HTP unknown)
					20.40%	Odani et al.[62]	from current cigarette users (HTP unknown) to ≥1mo cigarette quitter (HTP unknown)
					10.00%	Odani et al.[62]	from exclusive cigarette users to ≥6mo cigarette quitter (HTP unknown)
					18.60%	Odani et al.[62]	from exclusive cigarette users to ≥1mo cigarette quitter (HTP unknown)
			50-59	2019-21	8.50%	Odani et al.[62]	from current cigarette users (HTP unknown) to ≥6mo cigarette quitter (HTP unknown)
					18.00%	Odani et al.[62]	from current cigarette users (HTP unknown) to ≥1mo cigarette quitter (HTP unknown)
					7.90%	Odani et al.[62]	from exclusive cigarette users to ≥6mo cigarette quitter (HTP unknown)
					16.10%	Odani et al.[62]	from exclusive cigarette users to ≥1mo cigarette quitter (HTP unknown)
			60-74	2019-21	14.20%	Odani et al.[62]	from current cigarette users (HTP unknown) to ≥6mo cigarette quitter (HTP unknown)

					20.60%	Odani et al.[62]	from current cigarette users (HTP unknown) to ≥1mo cigarette quitter (HTP unknown)
					14.60%	Odani et al.[62]	from exclusive cigarette users to ≥6mo cigarette quitter (HTP unknown)
					22.90%	Odani et al.[62]	from exclusive cigarette users to ≥1mo cigarette quitter (HTP unknown)
		Men	20-74	2019-21	9.60%	Odani et al.[62]	from current cigarette users (HTP unknown) to ≥6mo cigarette quitter (HTP unknown)
					19.80%	Odani et al.[62]	from current cigarette users (HTP unknown) to ≥1mo cigarette quitter (HTP unknown)
					10.70%	Odani et al.[62]	from exclusive cigarette users to ≥6mo cigarette quitter (HTP unknown)
					20.60%	Odani et al.[62]	from exclusive cigarette users to ≥1mo cigarette quitter (HTP unknown)
		Women	20-74	2019-21	13.50%	Odani et al.[62]	from current cigarette users (HTP unknown) to ≥6mo cigarette quitter (HTP unknown)
					26.80%	Odani et al.[62]	from current cigarette users (HTP unknown) to ≥1mo cigarette quitter (HTP unknown)
					13.10%	Odani et al.[62]	from exclusive cigarette users to ≥6mo cigarette quitter (HTP unknown)
					26.10%	Odani et al.[62]	from exclusive cigarette users to ≥1mo cigarette quitter (HTP unknown)
**Dual users**	**Cigarette users**	Both	20+	2018-19	11.00%	Jones et al.[61]	from dual users to exclusive cigarette users
	**Dual Users**	Both	20+	2018-19	78.20%	Jones et al.[61]	from dual users to dual users
	**HTP users**	Both	20+	2018-19	7.10%	Jones et al.[61]	from dual users to exclusive HTP users
	**No use**	Both	20-74	2019-21	9.90%	Odani et al.[62]	from dual users to ≥6mo cigarette quitter (HTP unknown)
					21.20%	Odani et al.[62]	from dual users to ≥1mo cigarette quitter (HTP unknown)
				2020-21	14.90%	Odani et al.[62]	from dual users to ≥6mo cigarette quitter (HTP unknown)
					28.50%	Odani et al.[62]	from dual users to ≥1mo cigarette quitter (HTP unknown)
				2019-20	5.70%	Odani et al.[62]	from dual users to ≥6mo cigarette quitter (HTP unknown)
					14.90%	Odani et al.[62]	from dual users to ≥1mo cigarette quitter (HTP unknown)
			20+	2018-19	3.60%	Jones et al.[61]	from dual users to no use (no cig and no HTP)
			20-29	2019-21	9.60%	Odani et al.[62]	from dual users to ≥6mo cigarette quitter (HTP unknown)
					28.50%	Odani et al.[62]	from dual users to ≥1mo cigarette quitter (HTP unknown)
			30-39	2019-21	8.60%	Odani et al.[62]	from dual users to ≥6mo cigarette quitter (HTP unknown)

					19.90%	Odani et al.[62]	from dual users to ≥1mo cigarette quitter (HTP unknown)
			40-49	2019-21	8.10%	Odani et al.[62]	from dual users to ≥6mo cigarette quitter (HTP unknown)
					22.20%	Odani et al.[62]	from dual users to ≥1mo cigarette quitter (HTP unknown)
			50-59	2019-21	9.20%	Odani et al.[62]	from dual users to ≥6mo cigarette quitter (HTP unknown)
					20.00%	Odani et al.[62]	from dual users to ≥1mo cigarette quitter (HTP unknown)
			60-74	2019-21	13.80%	Odani et al.[62]	from dual users to ≥6mo cigarette quitter (HTP unknown)
					18.10%	Odani et al.[62]	from dual users to ≥1mo cigarette quitter (HTP unknown)
		Men	20-74	2019-21	8.60%	Odani et al.[62]	from dual users to ≥6mo cigarette quitter (HTP unknown)
					19.10%	Odani et al.[62]	from dual users to ≥1mo cigarette quitter (HTP unknown)
		Women	20-74	2019-21	14.00%	Odani et al.[62]	from dual users to ≥6mo cigarette quitter (HTP unknown)
					27.50%	Odani et al.[62]	from dual users to ≥1mo cigarette quitter (HTP unknown)
**Current HTP users**	**Cigarette users**	Both	20+	2018-19	0.00%	Jones et al.[61]	from exclusive HTP users to exclusive cigarette users
	**Dual users**	Both	20+	2018-19	0.80%	Jones et al.[61]	from exclusive HTP users to dual users
	**HTP users**	Both	20+	2018-19	89.90%	Jones et al.[61]	from exclusive HTP users to exclusive HTP users
	**No use**	Both	20+	2018-19	2.90%	Jones et al.[61]	from exclusive HTP users to no use (no cig and no HTP)
**Former Cigarette users**	**Cigarette users**	Both	20-74	2019-21	11.40%	Odani et al.[62]	from former exclusive cigarette users (quit > 30 day) to current cigarette users (HTP unknown)
					10.20%	Odani et al.[62]	from former exclusive cigarette users (quit > 1yr) to current cigarette users (HTP unknown)
					25.80%	Odani et al.[62]	from former exclusive cigarette users (1yr > quit > 30 days) to current cigarette users (HTP unknown)
					13.10%	Odani et al.[62]	from former cigarette users (quit > 1 yr, HTP unknown) to current cigarette users (HTP unknown)
					22.60%	Odani et al.[62]	from former cigarette users (1 yr > quit > 30 days, HTP unknown) to current cigarette users (HTP unknown)
				2020-21	9.80%	Odani et al.[62]	from former exclusive cigarette users (quit > 30d) to current cigarette users (HTP unknown)
					11.40%	Odani et al.[62]	from former cigarette users (quit > 30d, HTP unknown) to current cigarette users (HTP unknown)
			20-74	2019-20	12.80%	Odani et al.[62]	from former exclusive cigarette users (quit > 30d) to current cigarette users (HTP unknown)
					16.10%	Odani et al.[62]	from former cigarette users (quit > 30d, HTP unknown) to current cigarette users (HTP unknown)
			18-73	2019-20	2.90%	Matsuyama and Tabuchi[69]	from former cigarette users (quit > 1yr, HTP unknown) to current cigarette users (HTP unknown)

					22.70%	Matsuyama and Tabuchi[69]	from former cigarette users (quit <1yr, HTP unknown) to current cigarette users (HTP unknown)
			15+	2017-18	6.50%	Hori, Tabuchi, and Kunugita[66]	from former cigarette users (HTP unknown) to exclusive cigarette users
				2015-16	5.90%	Hori, Tabuchi, and Kunugita[66]	from former cigarette users (HTP unknown) to exclusive cigarette users
			20-29	2019-21	16.80%	Odani et al.[62]	from former exclusive cigarette users (quit > 30d) to current cigarette users (HTP unknown)
					26.20%	Odani et al.[62]	from former cigarette users (quit > 30d, HTP unknown) to current cigarette users (HTP unknown)
			30-39	2019-21	19.80%	Odani et al.[62]	from former exclusive cigarette users (quit > 30d) to current cigarette users (HTP unknown)
					20.70%	Odani et al.[62]	from former cigarette users (quit > 30d, HTP unknown) to current cigarette users (HTP unknown)
			40-49	2019-21	11.80%	Odani et al.[62]	from former exclusive cigarette users (quit > 30d) to current cigarette users (HTP unknown)
					12.10%	Odani et al.[62]	from former cigarette users (quit > 30d, HTP unknown) to current cigarette users (HTP unknown)
			50-59	2019-21	10.10%	Odani et al.[62]	from former exclusive cigarette users (quit > 30d) to current cigarette users (HTP unknown)
					12.10%	Odani et al.[62]	from former cigarette users (quit > 30d, HTP unknown) to current cigarette users (HTP unknown)
			60-74	2019-21	8.00%	Odani et al.[62]	from former exclusive cigarette users (quit > 30d) to current cigarette users (HTP unknown)
					9.70%	Odani et al.[62]	from former cigarette users (quit > 30d, HTP unknown) to current cigarette users (HTP unknown)
		Men	20-74	2019-21	12.00%	Odani et al.[62]	from former exclusive cigarette users (quit > 30d) to current cigarette users (HTP unknown)
					12.10%	Odani et al.[62]	from former cigarette users (quit > 30d, HTP unknown) to current cigarette users (HTP unknown)
		Women	20-74	2019-21	11.20%	Odani et al.[62]	from former exclusive cigarette users (quit > 30d) to current cigarette users (HTP unknown)
					18.30%	Odani et al.[62]	from former cigarette users (quit > 30d, HTP unknown) to current cigarette users (HTP unknown)
		Both	20-74	2019-21	17.10%	Odani et al.[62]	from former cigarette users (quit > 30 days) and HTP users to current cigarette users (HTP unknown)

					16.80%	Odani et al.[62]	from former cigarette users (quit > 1 yr) and HTP users to current cigarette users (HTP unknown)
					19.60%	Odani et al.[62]	from former cigarette users (1yr > quit > 30 days) and HTP users to current cigarette users (HTP unknown)
				2020-21	13.40%	Odani et al.[62]	from former cigarette users (quit > 30 days) and HTP users to current cigarette users (HTP unknown)
				2019-20	20.10%	Odani et al.[62]	from former cigarette users (quit > 30 days) and HTP users to current cigarette users (HTP unknown)
			20-29	2019-21	34.20%	Odani et al.[62]	from former cigarette users (quit > 30 days) and HTP users to current cigarette users (HTP unknown)
			30-39	2019-21	21.50%	Odani et al.[62]	from former cigarette users (quit > 30 days) and HTP users to current cigarette users (HTP unknown)
			40-49	2019-21	12.50%	Odani et al.[62]	from former cigarette users (quit > 30 days) and HTP users to current cigarette users (HTP unknown)
			50-59	2019-21	14.00%	Odani et al.[62]	from former cigarette users (quit > 30 days) and HTP users to current cigarette users (HTP unknown)
			60-74	2019-21	13.20%	Odani et al.[62]	from former cigarette users (quit > 30 days) and HTP users to current cigarette users (HTP unknown)
		Men	20-74	2019-21	25.00%	Odani et al.[62]	from former cigarette users (quit > 30 days) and HTP users to current cigarette users (HTP unknown)
		Women	20-74	2019-21	13.20%	Odani et al.[62]	from former cigarette users (quit > 30 days) and HTP users to current cigarette users (HTP unknown)
	**Dual Users**	Both	15+	2017-18	1.80%	Hori, Tabuchi, and Kunugita[66]	from former cigarette users (HTP unknown) to dual users
				2015-16	0.20%	Hori, Tabuchi, and Kunugita[66]	from former cigarette users (HTP unknown) to dual users
	**HTP users**	Both	15+	2017-18	2.60%	Hori, Tabuchi, and Kunugita[66]	from former cigarette users (HTP unknown) to exclusive HTP users
				2015-16	0.50%	Hori, Tabuchi, and Kunugita[66]	from former cigarette users (HTP unknown) to exclusive HTP users
	**No use**	Both	15+	2017-18	89.10%	Hori, Tabuchi, and Kunugita[66]	from former cigarette users (HTP unknown) to no users (no cig and no HTP)
				2015-16	93.40%	Hori, Tabuchi, and Kunugita[66]	from former cigarette users (HTP unknown) to no users (no cig and no HTP)
